# Microbial ecology and biogeochemistry of hypersaline sediments in Orca Basin

**DOI:** 10.1371/journal.pone.0231676

**Published:** 2020-04-21

**Authors:** Lisa M. Nigro, Felix J. Elling, Kai-Uwe Hinrichs, Samantha B. Joye, Andreas Teske

**Affiliations:** 1 Department of Marine Sciences, University of North Carolina at Chapel Hill, Chapel Hill, NC, United States of America; 2 Center for Marine Environmental Sciences (MARUM), University of Bremen, Bremen, Germany; 3 Department of Marine Sciences, University of Georgia, Athens, GA, United States of America; University of Utah, UNITED STATES

## Abstract

In deep ocean hypersaline basins, the combination of high salinity, unusual ionic composition and anoxic conditions represents significant challenges for microbial life. We used geochemical porewater characterization and DNA sequencing based taxonomic surveys to enable environmental and microbial characterization of anoxic hypersaline sediments and brines in the Orca Basin, the largest brine basin in the Gulf of Mexico. Full-length bacterial 16S rRNA gene clone libraries from hypersaline sediments and the overlying brine were dominated by the uncultured halophilic KB1 lineage, Deltaproteobacteria related to cultured sulfate-reducing halophilic genera, and specific lineages of heterotrophic Bacteroidetes. Archaeal clones were dominated by members of the halophilic methanogen genus *Methanohalophilus*, and the ammonia-oxidizing Marine Group I (MG-I) within the Thaumarchaeota. Illumina sequencing revealed higher phylum- and subphylum-level complexity, especially in lower-salinity sediments from the Orca Basin slope. Illumina and clone library surveys consistently detected MG-I Thaumarchaeota and halotolerant Deltaproteobacteria in the hypersaline anoxic sediments, but relative abundances of the KB1 lineage differed between the two sequencing methods. The stable isotopic composition of dissolved inorganic carbon and methane in porewater, and sulfate concentrations decreasing downcore indicated methanogenesis and sulfate reduction in the anoxic sediments. While anaerobic microbial processes likely occur at low rates near their maximal salinity thresholds in Orca Basin, long-term accumulation of reaction products leads to high methane concentrations and reducing conditions within the Orca Basin brine and sediments.

## Introduction

**Fig 1:**
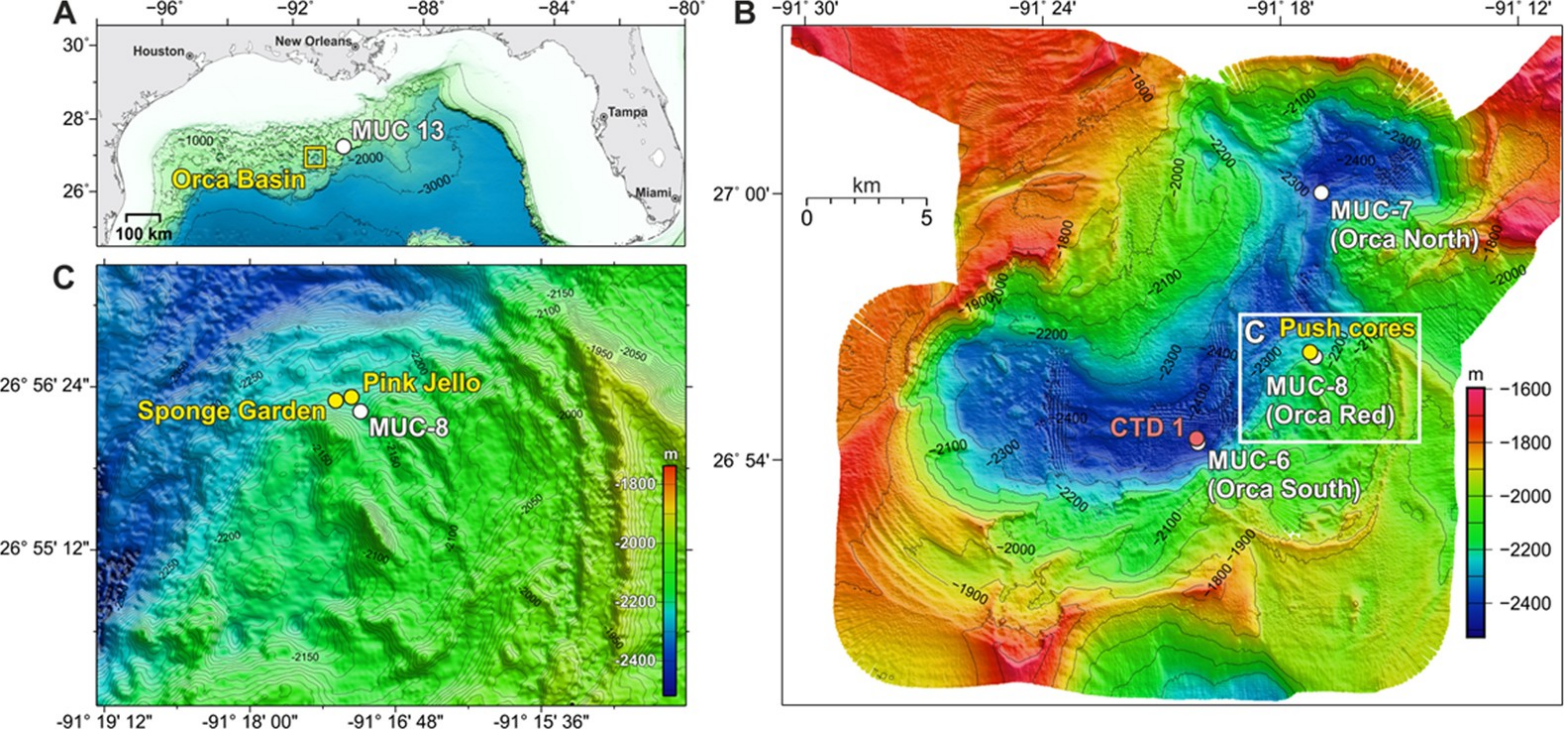
Sampling site locations. A. Location of Orca Basin in the Gulf of Mexico. B. Sampling map within Orca Basin displaying multicore locations (white) and the south sub-basin CTD location (red). C. The region indicated by the white box in B shows the location of DSV Alvin push cores from expedition AT18-02. The map was created with the GEBCO Compilation Group GEBCO 2019 Grid (doi:10.5285/836f016a-33be-6ddc-e053-6c86abc0788e).

The Orca Basin is a large deep hypersaline anoxic basin (DHAB) located in a seafloor depression along the Texas-Louisiana continental slope (26°56’N, 91°19’W). The brine fills two adjacent sub-basins that are separated by a shallow saddle and extends from ca. 2200 m water depth to the basin floor at approx. 2400 m depth [1] (Fig 1). Unlike hypersaline basins in the Red Sea and Mediterranean Sea, which are mostly influenced by divergent and convergent plate tectonics, Orca Basin lies on a passive continental margin where a previously buried rock salt layer breached the seafloor and started leaching brine that accumulates in adjacent seafloor basins [2]. The onset of anoxic conditions in the brine and underlying sediment, dated at 7900 ± 170 years ago using radiocarbon, is visible in the sediments as a transition from underlying grey mud typical for oxygen-exposed sediments on the open slope to overlying black mud containing metal sulfides that indicate anoxia [3]. Reflecting its origin from predominantly halite dissolution, the brine was enriched by factor of 7.5 and 8.5 in Na^+^ and Cl^-^, respectively, whereas it is only moderately enriched by factors 1.3 to 2.6 in K^+^, Ca^2+^, and SO_4_^2-^, and slightly depleted in Mg^2+^ [1,4].

The strong density gradient across the brine/seawater interface in the Orca Basin prevents advective mixing, maintaining anoxia in the brine and the underlying sediments [1,4]. The brine-seawater interface also serves as a particle trap, where total suspended matter accumulates to concentrations as high as 880 μg/l, eight times higher than the overlying seawater [5,6]. In comparison to seawater, the brine is slightly acidic (pH = 6.5). Microbial activity is concentrated at the brine-seawater interface [7], which is congruent with microbial consumption of the electron acceptors nitrate and oxygen. The brine is enriched in biogenic compounds such as the products of microbial remineralization of organic matter, silica and phosphate, as well as ammonium and methane [1,4,8].

Within the black and grey-colored Orca Basin sediments, porewater salinities averaged 260 ppt (100 cm downcore) reflecting the overlying brine, and after that gradually decreased with depth [9]. In the hypersaline sediments, predominantly marine-derived organic matter is degraded slowly and has a long residence time [10,11]. At depth, buried fronds of *Sargassum* have been found intact, only partially degraded [12], indicating attenuated microbial activity in the hypersaline sediments. Porewater geochemical data revealed high sulfate concentrations that reached ~44 to 55 mM at the sediment surface and decreased slowly downcore [9]. Porewater sulfide concentrations up to ~150 μM were detected in hypersaline cores [9], but sulfide was not detectable in the deep brine [4]. Solid-phase sulfur compounds were identified predominantly as FeS in black sediments, and pyrite in the gray sediments [13]. Sediments from the relatively oxidized, non-sulfidic saddle appeared brown with red laminations, [9,14] resulting from high concentrations of iron oxide in the form of hematite [13].

In line with similar observations in deep brine lakes of the Mediterranean and the Red Sea [15], microbial studies in Orca Basin have demonstrated microbial activity and population peaks within the oxycline and halocline. Measurements of adenosine 5’-triphosphate and uridine uptake indicated a highly active microbial community within an approx. 50 m thick layer (2173 to 2221 m) where oxygen decreased from approx. 71 to 13% of deep seawater concentrations, while microbial activity in the deep brine decreased substantially [16]. Archaeal isoprenoid lipids displayed a distinct peak approximately 2260 to 2270 m depth, indicating that archaea thrive or at least accumulate in this deep halocline horizon [17]. Culturable iron- and manganese-oxidizing and reducing bacteria showed relative abundance peaks above the steepest halocline layer near 2170 and 2220 m and 2230 to 2250 m, respectively [18]. Microbial community surveys of Orca Basin sediments are so far limited to archaeal 16S rRNA gene sequences of a few sediment samples [19]. As a first step toward a comprehensive microbial census of Orca Basin, we geochemically characterized sediment cores from the deep brine basins and slope sediments, and analyzed the bacterial and archaeal community composition of these sediments based on clone libraries of nearly full-length 16S rRNA genes as well as Illumina sequencing of partial 16S rRNA genes.

## Materials and methods

### Sample collection and processing

**Fig 2:**
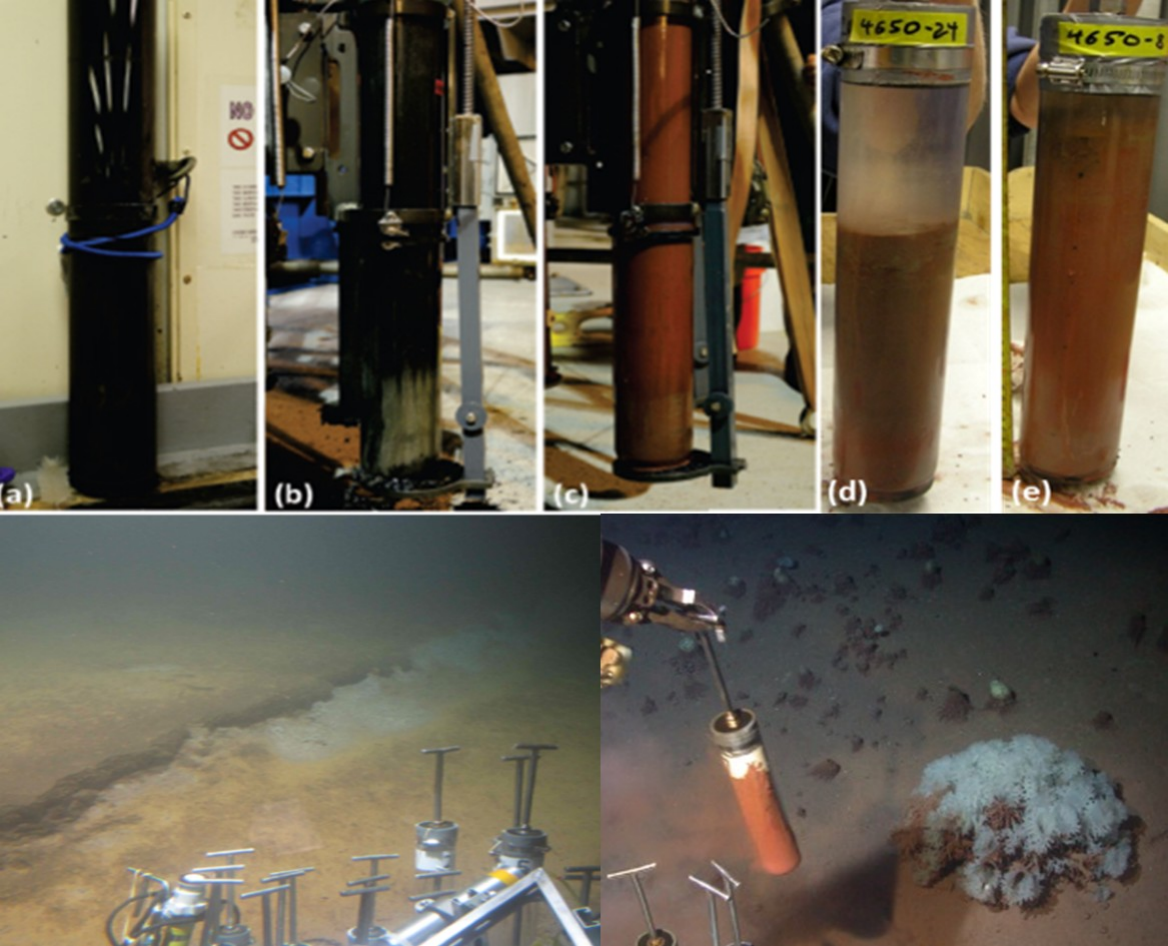
Sediment core characteristics. Top row, photographs of Orca Basin multicores taken from (a) black sediment in the south sub-basin (MUC 6), (b) dark-grey sediment in the north sub-basin (MUC 7), and (c) red sediments of the basin slope (MUC 8). Push cores 4650–24 (d) and 4650–8 (e) were obtained with HOV *Alvin* from the basin slope near the saddle region. Bottom row, *Alvin* photographs of the coring sites for Pink Jello core 4650–24 at 2198 m depth (left) and for Sponge Garden core 4650–8 at 2167 m depth (right; the core being collected on this snapshot is 4560–7, next to 4560–8).

Sediment core samples were collected on R/V *Atlantis* during expedition AT18-2 in November 2010. A collection permit was acquired by the Bureau of Ocean Energy Management (BOEM OCS Notice NG14-002). A shipboard multicorer was used to obtain sediment cores of approximately 70 cm length from the anoxic brine-filled south and north sub-basins (MUC-6, depth 2432 m, collected on Nov. 18, 2010, at 26°54.48N and 91°20.09W, and MUC-7, depth 2339 m, collected on Nov. 19, 2010, at 27°00N and 91°16.99W), and from an elevated ridge, or saddle, separating the N and S basins in the Orca Basin (MUC-8, also called the Red Ridge core due to the color of the sediments, collected on Nov. 20, 2010, in 2180 m depth at 26°56.25N and 91°17.10W). Sediment push cores were collected by submersible *Alvin* during dive 4650 from the steep flanks of Orca Basin near the oxycline and halocline. Based on site and sediment characteristics, these *Alvin* cores were subsequently called Pink Jello cores (core 4650–24 for microbiology, and adjacent core 4650–19 for geochemistry; depth 2198 m) and Sponge Garden cores (core 4650–4 for microbiology, adjacent core 4650–8 for geochemistry; depth 2167 m) (Fig 2). A background core representing deep Gulf of Mexico slope sediment unaffected by brine seepage (MUC 13) was collected from a depth of 2068 m on Nov. 27, 2010, at 27°07.41N and 90°17.27W. For molecular analysis, brine from the south sub-basin was collected using Niskin bottles attached to a rosette and equipped with a CTD.

Sediment cores were immediately capped after shipboard recovery, and stored at 4°C until they were processed within 12 hours of collection. Overlying water was removed with a sterile syringe. Multicorer sediment cores were divided into 5-cm sections, while push cores were sectioned in 1 to 5-cm intervals. All materials used for core extrusion were either autoclaved or ethanol-washed prior to sampling. Water for molecular analysis was transferred from Niskin bottles into sterile containers; subsequently, approximately 500 mL was vacuum-filtered onboard using a sterile 47-mm diameter polycarbonate filter with 0.22 μm pore size. All samples for molecular analyses were immediately frozen at -80°C after processing.

### Sample preparation for geochemical analysis

Geochemistry subsamples were collected from each *Alvin* push core and each multicore during core sectioning. A 5-ml sediment sample for use in determining methane concentration and carbon isotopic composition (δ^13^C-CH_4_) was mixed immediately with 2 ml of 1 M NaOH solution in a 30-ml serum vial; vials were capped with thick butyl rubber stoppers and crimp-sealed. For multicore sulfate (SO_4_^2-^) and sulfide (H_2_S) measurements, porewater samples were obtained by centrifuging 50-ml of sediment at 3000 rpm for 15 minutes. Samples were pushed through a 0.22-μm syringe filter into sterile falcon tubes. For sulfate, 1 ml of porewater was added to 50 μl of 50% concentrated (6N) HCl in a 2 ml microcentrifuge tube. Each tube was bubbled with N_2_ for three minutes to remove sulfide, and samples were stored at 4°C until analysis by ion chromatography.

Sulfide concentration was determined by aliquoting 1 ml of porewater into 0.1 ml of 0.1 M zinc acetate solution in a 2-ml microcentrifuge vial; samples were mixed and stored subsequently at 4°C until further analysis. The remaining porewater was stored at -20°C for salinity measurements. For hydrogen (H_2_) concentration determination, a 3-mL sediment sample was collected into a cut-off syringe and transferred to a helium-purged serum vial, which was capped with a rubber septum and crimp-sealed. Vials were purged with helium and then incubated to allow headspace equilibration at 4°C for four days before analysis [20].

*Alvin* pushcores processed for geochemical analyses were extruded and sliced in 3-cm sections, and dissolved gas and porewater samples were analyzed as described previously [21]. Methane samples were obtained and preserved by transferring 5 mL of wet sediment into a 12-mL He-flushed serum vial amended with 4 mL of helium-purged 2 M NaOH, capping the vial with a butyl rubber septum, crimp-sealing, shaking and storing samples upside down at room temperature until analysis. The remaining sediment was then transferred to Ar-flushed squeezer cups, then porewater was collected into an Ar-purged syringe, and passed through a 0.22-μm Target® filter before being aliquoted for different analyses. Porewater subsamples for cation (Na^+^, K^+^, Mg^2+^) and anion (Cl^-^, SO_4_^2-^) concentration determination were preserved with 1 μM (final concentration) of trace metal grade nitric acid. To preserve subsamples for sulfide concentration analysis, 0.5 to 2 mL of pore water was transferred to vials containing 1 mL of 20% (w:w) zinc acetate. The remaining filtered pore water (without nitric acid or zinc sulfate addition) was either analyzed immediately for ammonium (NH_4_^+^) or frozen until processed for dissolved organic carbon (DOC) and salinity.

### Geochemical measurements

The salinity of Multicore pore water and overlying brine was determined in ppt [g salt/kg water] with an Atago Master-S / Millα salinity refractometer after being filtered through a 0.45-μm syringe filter and diluted by weight with deionized water for high salinity samples that were out of range of the instrument. Methane concentrations from multicore sediment samples were analyzed by injecting 1 mL of headspace gas into a ThermoFinnigan TraceGC gas chromatograph equipped with a flame ionization detector. Methane and other hydrocarbons were separated isothermally at 40°C on a Carboxen-1006 PLOT fused-silica capillary column (0.32 mm × 30 m; Supelco Inc., USA). An independently-certified methane standard (Scott Specialty Gases®) was used for calibrating the concentration measurements. Methane samples from *Alvin* push core sediments were measured with a Shimadzu gas chromatograph as described previously [21]. Porewater concentrations of methane were calculated from headspace concentrations, sediment volume, sediment porosity (determined gravimetrically), temperature and pressure.

Analysis of δ^13^C-CH_4_ values was performed on a gas chromatography combustion isotope ratio mass spectrometry (GC–C-IRMS) system combining a ThermoFinnigan Trace GC Ultra with a DELTA Plus XP mass spectrometer via a ThermoFinnigan GC Combustion III interface, as described previously [22]. Hydrogen samples were measured on the ship with a Peak Performer 1 Reduction Gas Analyzer equipped with mercury oxide detector (detection limit 800 parts per trillion; Peak Laboratories, USA). Calibrations were performed with a 1% H_2_ standard balanced in He (Scott Specialty Gases, USA). Repeated measurements were performed with a standard to determine the precision (<1.5%). Porewater H_2_ concentrations were calculated from headspace concentrations and the solubility constant for H_2_ corrected for temperature and salinity [23], as described previously [24]. Push core DIC measurements were made on samples preserved and stored under a helium atmosphere until quantification with infrared detection on a Shimadzu TOC® analyzer [21]. Multicore porewater DIC samples were prepared by freezing −20°C until analysis for concentration and δ13C by injecting 1 mL headspace gas after acidification with 0.2 mL of 0.1 M phosphoric acid into a Hewlett–Packard 5890 GC equipped with a 6 m Poroplot Q column held at 35°C and a Finnigan Mat DELTA S IRMS [19].

DOC, phosphate, and major cations and anions were measured as previously described [21], after diluting samples with high salt content. Sulfide concentrations were measured colorimetrically [25]. NH_4_^+^ concentrations were measured by the indo-phenol method [26]. For the multicorer deployments, some of the geochemical measurements (cations and anions, DOC and phosphate) were obtained from a different core than the core used for microbial 16S rRNA gene sequencing and the remaining geochemical measurements.

### DNA extraction and sequencing

Sediment DNA for Sanger sequencing was extracted with the PowerSoil DNA isolation kit according to the manufacturer’s instructions (MoBio, Carlsbad, CA) except triplicate ~0.5 g of sediment from each sample was centrifuged at maximum speed (16000 x g) for 5 minutes and overlying water decanted before processing. After bead beating, the supernatants from three tubes of the same sample were combined into one spin filter column, and the final eluate was reduced to 50 μL. For the brine sample, DNA was extracted from the filter with the PowerSoil DNA isolation kit according to the manufacturer’s recommendations, except the final volume was adjusted to 50 μL. The bacterial and archaeal 16S rRNA genes were amplified with Qiagen’s HiFidelity polymerase according to the manufacturer’s recommendations for buffers, polymerase, and Q solution. In addition, MgCl_2_ was used at a final concentration of 2 mM. Primers B8f (5′-AGRGTTTGATCCTGGCTCAG-3′) and B1492R (5′-CGGCTACCTTGTTACGACTT-3′) for bacterial 16S rRNA genes and A8f (5′-TCCGGTTGATCCTGCC-3′) and A1492 (5′-GGCTACCTTGTTACGACTT-3′) for archaeal 16S rRNA genes [27] were used at a final concentration of 2 μM each. Amplification was performed in a thermal cycler under the following conditions: an initial denaturation 95°C for 5 min, 35 cycles of 95°C for 30 s, 52°C for 1 min and 72°C for 2 min, and a final extension at 72°C for 10 min. PCR products were electrophoresed on a 1% agarose gel amended with GelRed (Biotum, Hayward, CA) and visualized under UV light. PCR products were purified with the minElute PCR Purification Kit or, if multiple banding was observed, with the MinElute Gel Extraction kit (Qiagen, Valencia, CA). Bacterial 16S rRNA gene PCR products were ligated to the TOPO TA PCR 2.1 vector (Invitrogen, Carlsbad, CA) according to the manufacturer’s instructions, with the exception that reactions were incubated for 16 hours. Archaeal 16S rRNA gene PCR products were ligated to the pDrive cloning vector (Qiagen) according to the manufacturer’s recommendations, at a ligation temperature of 8°C. All vector reactions were transformed into *Escherichia coli* TOP10 chemically competent cells (Invitrogen, Carlsbad, CA) according to the manufacturer’s instructions. Transformed bacteria were plated on LB agar with 50 μg/mL kanamyacin and 40 μg/mL X-gal. White colonies were arbitrarily selected, picked and re-streaked on another LB agar plate for bidirectional Sanger sequencing using vector primers M13 F and M13 R (Genewiz, South Plainfield, NJ).

Illumina sequencing samples were thawed on ice and 0.5 g of sediment of each sample was collected using a 1-mL syringe and placed in a 96-well MoBio bead-beating plate for subsequent extraction with the Powersoil-htp 96 Well DNA Isolation Kit (MoBio, Carlsbad, CA). DNA extraction, 16S rRNA gene amplification of the V4 fragment using bacterial/archaeal primers 515F and 806R, and sequencing on the Miseq2000 platform was performed at Joint Genome Institute (Walnut Creek, CA) in 2012 according to the Earth Microbiome Project standard protocol [28]. For illumina sequencing, the primer 515F (5’-GTGCCAGCMGCCGCGGTAA-3’) is extended in 5’-direction by a 5’-illumina adapter, forward linker pad and forward primer linker; the reverse primer 806R (5’-GGACTACHVGGGTWTCTAAT-3’) is extended by a reverse complement of 3’-illumina adapter, Golay barcode, reverse primer pad and reverse primer linker [28].

### Sequencing analysis

Sanger sequence reads were assembled and edited in Sequencher (Genecodes, Ann Arbor, MI). Sequences were aligned with SINA 1.2.11 [29] and were imported into ARB [30] and manually corrected. A maximum likelihood tree was constructed using RaxML in ARB. Statistical support of nodes was assessed by 1000 bootstrap replicates, using the rapid bootstrap analysis method as implemented in ARB.

Illumina sequences were analyzed with QIIME v1.8 [31]. Sequence quality scores were plotted and sequences truncated at a cutoff of quality score of 25. Paired ends were joined with the fastq-join method [32] and sequence data demultiplexed and identified by the 12 base Golay error correcting barcodes (attached to the 806R primer) utilizing the split libraries script in QIIME. Sequences were grouped into operational taxonomic units (OTUs) at 97% similarity and putative chimeras were removed with UPARSE [33]. Singletons were removed before further analysis. Taxonomic classifications for OTU sequences were determined using QIIME’s UCLUST approach and SILVA reference and taxonomic mapping files of SILVA release 111 [34]. Updated taxonomic names for candidate division phyla were also included in the figure (SILVA v132). Sequences were aligned with the Python implemented NAST alignment algorithm [35]. Phylogenetic trees relating OTUs were constructed using the FastTree method [36]. Alpha diversity was calculated as Shannon diversity [37] and Faith’s PD whole tree [38] metrics based on multiple rarefactions of the data. Beta diversity was estimated using weighted Unifrac [39] and visualized with Principal Coordinate Analysis (PCoA) at a rarefaction depth of 22600 sequences using the phyloseq package in R [40].

### Sequence accession numbers

Genbank accession numbers of the nearly full-length 16S rRNA gene sequences are KP204602-KP204843, KR857575-KR857679, KT223145-KT223310 and KY563090-KY563092. The Illumina Miseq dataset is deposited in the NCBI Short Read Archive under the SRA Study number SRP137375.

## Results

### Sediment characteristics

The sediment core from the south sub-basin (MUC 6) was black over its entire length, and the north sub-basin multicore (MUC 7) was black with a transition to dark grey starting at 45 cm depth (Fig 2). The multicore from the saddle area (MUC 8) was red with brownish-red laminations throughout the core (Fig 2). In all three cases, the cores over-penetrated the extremely porous, fine-grained sediments, and neither the sediment surface layer nor overlying brine was recovered. Therefore, the exact sediment depths remain unknown and the true sediment water interface is not represented by these data. The background continental slope multicore contained brown sediment more typical for the Gulf of Mexico. The sediment push cores collected by *Alvin* at 2167 m depth in the Orca Basin edge recovered conspicuous rosy-red sediments; this area also harbored abundant glass sponges. The basin edge sediments collected by *Alvin* at 2198 m depth showed a jelly-like consistency and were characterized by reddish-brown laminations; this area did not harbor glass sponges (Fig 2). Unlike the Orca Basin multicores, the continental slope multicore and the *Alvin* push cores were not over-penetrated and contained overlying water.

### Geochemical characteristics

#### Salinity and major anions and cations

Salinity estimates were similar to values previously reported in Orca Basin. Refractometer salinity estimates indicated that the sediment porewater salinity matched that of the overlying brine. Porewater salinity of sediments in the south sub-basin decreased from 267 ppt at the top of the core to 261 ppt at 60 cm depth. The salinity of the north sub-basin brine was 258 ppt, almost identical to the previously determined salinity value of 258.1 ppt for Orca Basin brine [1]. Sediment pore water salinity in the north sub-basin decreased from 255 ppt at the top of the core to 234 ppt at the bottom of the core. In the Red Ridge MUC core, porewater salinity ranged from 53 to 59 ppt, confirming the position of this sampling location along the halocline.

**Fig 3:**
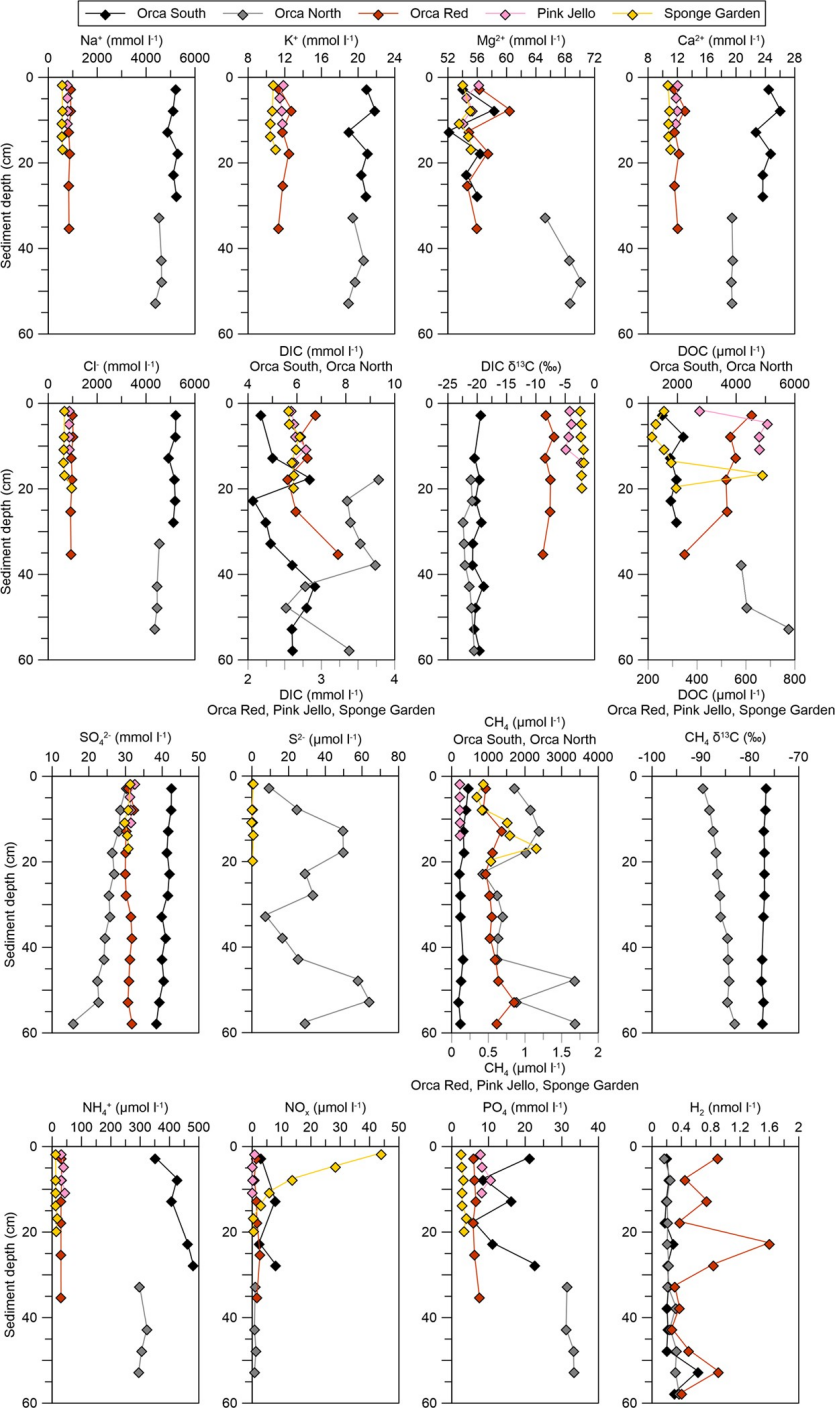
Porewater geochemical measurements in Orca Basin. Geochemical measurements are plotted as a function of sediment depth (cm below surface). Black diamonds, Orca south sub-basin (MUC-6); grey diamonds, Orca north sub-basin (MUC-7); red diamonds, Red Ridge core (MUC-8); pink diamonds, Pink Jello core (Alvin core 4560–19), yellow diamonds, Sponge Garden core (Alvin core 4560–8). Multicore samples were sectioned in 5 cm intervals, while push core samples were sectioned in 3 cm intervals. Some sites do not have complete geochemical gradient data due to limited sample material.

Porewater major anions and cations indicated little concentration change with depth (Fig 3). Concentrations of Na^+^ and Cl^-^ were elevated relative to seawater, approximately 5 M in south sub-basin sediments and 4.5 M in north sub-basin sediments. Compared to the continental slope background site core, Na^+^ and Cl^-^ in the Red Ridge multicore and the Pink Jello core were elevated by a factor of 1.5. The Sponge Garden geochemistry core exhibited Na^+^ and Cl^-^ concentrations similar to seawater. In the north and south sub-basin cores, K^+^ and Ca^2+^ concentrations were about double seawater concentrations (Fig 3).

#### Porewater sulfate and sulfide

In the north sub-basin MUC core, porewater sulfate [SO_4_^2-^] concentrations decreased slightly with depth, from near 30 mM in the 0-5-cm section to 23 mM at 45–50 cm depth. In the south sub-basin core, porewater sulfate concentrations started at 43 mM at 0–5 cm and decreased slightly downcore to 39 mM at 55–60 cm depth, consistent with previous studies [9,14]. The concentration of sulfate in Orca Basin brine is roughly 46 mM [1,4]; the lower sulfate concentrations in the MUC cores likely resulted from over-coring and loss of surficial sediment layers where sulfate concentrations may have been higher. Porewater sulfide concentrations remained below the detection limit (<3 μM) in the south sub-basin and ridge sediment cores, and contrasted with detectable sulfide (7 to 64 μM) in the north sub-basin MUC core.

Porewater methane concentration ranged from 91 to 156 μM in the south sub-basin and from 565 to 1400 μM in the north sub-basin core. The low δ^13^C-CH_4_ values, -75.5 to -76.7‰ in the south sub-basin and -83.1 to -89.6‰ in the north sub-basin core, indicated a biogenic source for methane in these hypersaline sediments [41]. The observed δ^13^C-CH_4_ values were similar to those measured (-72.6 to -75.7 ‰) previously in deep sediment cores of the north sub-basin (Site 618) that were obtained through the Deep-Sea Drilling Program [42].

#### Porewater dissolved carbon and ammonium

Porewater concentrations of dissolved inorganic carbon (DIC) fluctuated between 4 and 7 mM in the south sub-basin core, and between 5.5 to 9.5 mM in the north sub-basin core; the DIC concentrations of the ridge and slope cores clustered around 5.5 and 7 mM (Fig 3). All measured porewater DIC concentrations were clearly above the DIC concentration (4.5 mM) of the brine [43] and therefore indicated microbial mineralization of organic matter. However, DIC concentrations remained quite variable and did not increase consistently over the length of the cores (Fig 3). The δ^13^C-DIC values of porewater from the Red Ridge, Pink Jello, and Sponge Garden cores varied between -2‰ and -10‰, whereas the δ^13^C-DIC values of the hypersaline cores were near -20‰ (Fig 3) [19].

Porewater concentrations of dissolved organic carbon (DOC) in the Orca Basin sediments reached approx. 5800 μM in north sub-basin sediment and 2200 μM in south sub-basin sediment, and were highly elevated compared to the overlying brine (320 to 350 μM DOC) [7]. In contrast, porewater DOC concentrations of ca. 200 to 700 μM were observed in the Red Ridge core and in the Sponge Garden and Pink Jello cores (Fig 3). Porewater NH_4_^+^ concentrations were generally high in the hypersaline cores, up to 480 μM in south sub-basin sediment and 323 μM in north sub-basin sediment; in contrast, they remained below 50 μM in non-hypersaline sediments (Fig 3). Sediment NH_4_^+^ concentrations in the south sub-basin were close to previously reported concentrations of 500 μM in the Orca Basin brine [8].

### Bacterial 16S rRNA gene clone libraries

**Fig 4:**
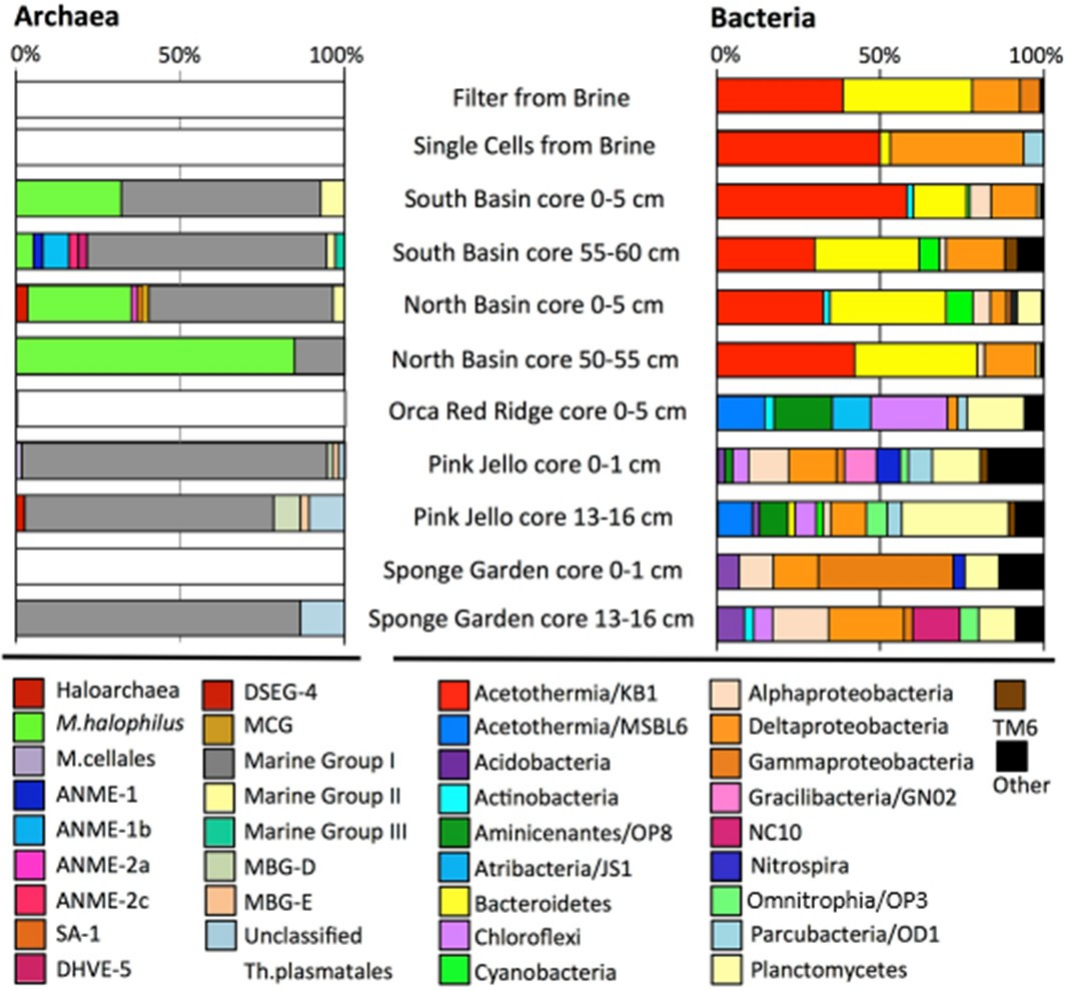
Profiles of archaeal and bacterial 16S rRNA gene clone library sequences for Orca Basin sediment and brine samples. The bar labeled “Single Cells from Brine” shows results for single cells isolated directly from the brine on a separate research expedition, and identified by cell sorting and partial 16S rRNA gene sequencing [44].

**Fig 5:**
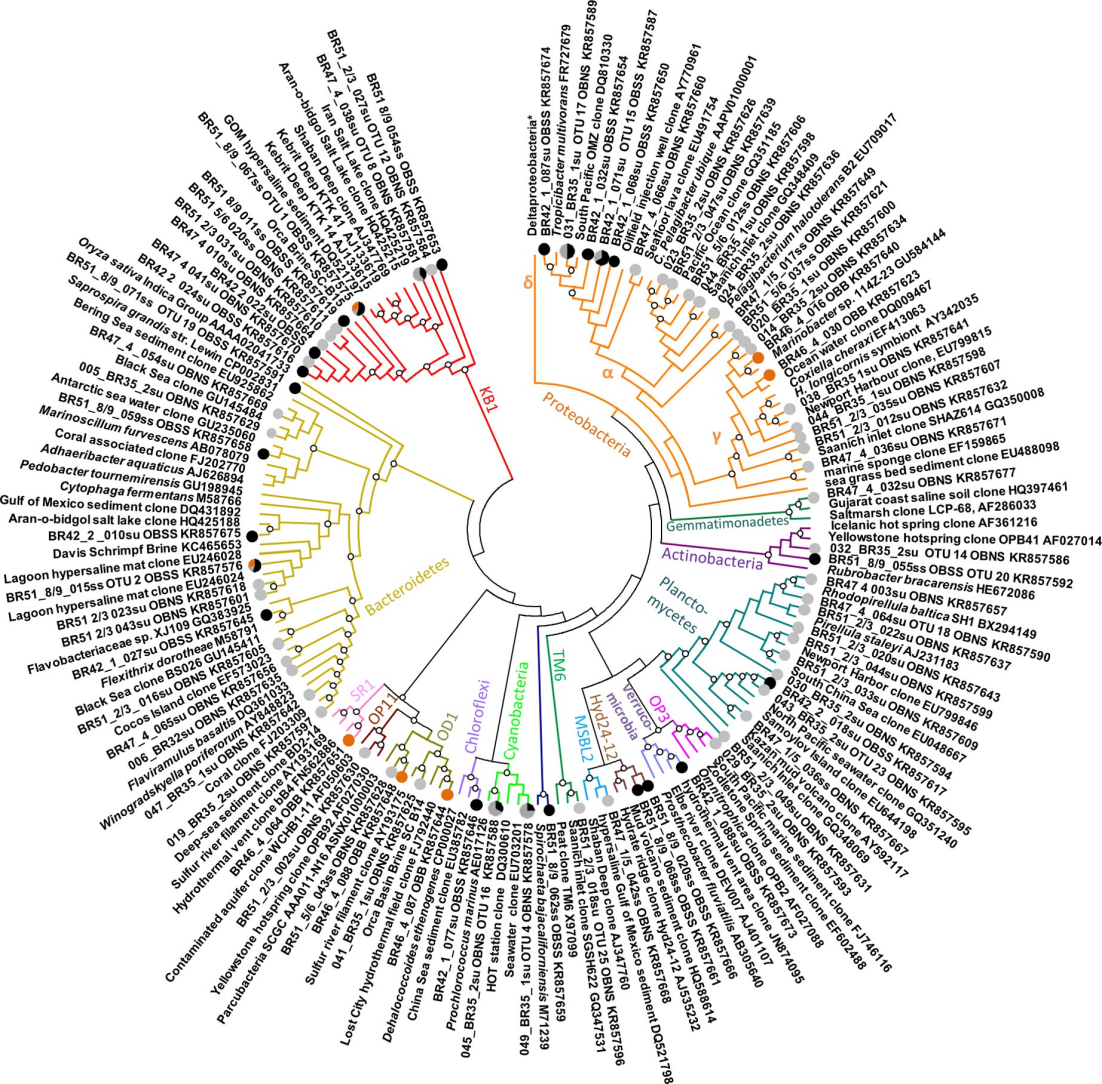
Radial phylogeny of Orca Basin bacterial 16S rRNA gene clone library sequences. Trees were constructed with RaxML. Nodes are annotated with bootstrap numbers from 1000 replicates; only bootstrap numbers above 50% are shown. Deltaproteobacteria and Archaea are resolved further in detailed phylogenies (S1 and S2 Figs). Sequences from south sub-basin sediments (black circles), north sub-basin sediment (grey circles), and Orca Basin brine (orange circles) are shown.

The bacterial 16S rRNA gene sequences from the deep anoxic brine and brine sediments represented mostly the Deltaproteobacteria, Candidate Division KB1, and Bacteroidetes, with partial OTU overlap between these habitats (Figs 4 and 5). Interestingly, sequences affiliated with these three phylum or subphylum-level groups showed consistent affinities to cultured halophilic species and genera, or to uncultured phylotypes from hypersaline habitats. The taxonomic associations of clone library 16S rRNA gene sequences from the filtered deep brine sample (Fig 4) were consistent with 16S rRNA gene sequences from a cell-sorted sample from a different Orca Basin research expedition [44]. Bacteroidetes sequences from the Orca Basin most closely resembled those found in other hypersaline habitats, in particular a hypersaline microbial mat in the Candeleria lagoon of Puerto Rico [45] and an evaporitic crust community from the salt evaporation ponds in Guerrero Negro, Mexico [46].

The most frequently recovered Sanger sequencing phylotypes in the deep brine and in hypersaline sediments were members of the uncultured Candidate Division KB1. This monophyletic lineage was originally detected by 16S rRNA gene sequencing in the hypersaline Red Sea basin Kebrit Deep [47], and was noted subsequently in high salinity environments [15). A partial single-cell genome of KB1 from Orca Basin showed that its proteins were adapted to high salinity; the genome also encoded an uptake system for the osmolyte glycine betaine, which could also be used as a carbon and energy source [44], supporting ^14^C incorporation experiments from Medee brine samples [48].

Deltaproteobacterial sequences (S1 Fig) were related to members of sulfate-reducing halophilic genera such as *Desulfocella halophila* [49] and some constituted the closest sister lineages to cultured halophiles (*Desulfohalobium retbaense*, *Desulfovermiculus halophilus*, *Desulfohalobium utahense*, and *Desulfosalsimonas propionicica)* [50–53]. These halophilic bacteria can respire sulfate, thiosulfate, sulfite, and elemental sulfur and they use diverse energy sources, including low-molecular weight organic acids, fatty acids, alcohols, and amino acids. The Orca Basin Deltaproteobacteria were also related to clones from hypersaline sites, including a Mediterranean solar saltern (NCBI accession FJ536432) and the brine-seawater interface of the hypersaline basin Shaban Deep in the Red Sea [54] (S1 Fig).

Near full-length 16S rRNA gene sequences related to the SEEP-SRB1 group within the Desulfobacteriaceae [55] were found in all Orca Basin cores, on the slope and in both of the brine basins (S1 Fig). SEEP-SRB1 sequences were particularly abundant in the south sub-basin core at 50–55 cm, comprising 23.4% of all deltaproteobacterial sequences. However, no sequences could be confidently assigned to the SEEP-SRB 1a subgroup, the dominant syntrophic partners of anaerobic methane oxidizing archaea (ANME) [56]. Two other deltaproteobacterial groups have been reported to be physically associated with ANME archaea, the SEEP-SRB2 cluster [57] and sequences related to *Desulfobulbaceae* [58], but neither were identified in the clone libraries.

The clone library sequences from the non-hypersaline sediments represented more diverse bacterial groups (Fig 4); their OTUs were entirely distinct from their hypersaline counterparts. Bacterial phylotypes in the Red Ridge multicore were dominated by the uncultured candidate divisions OP8, comprehensively described as candidate phylum Aminicenantes [59], and JS1, comprehensively described as candidate phylum Atribacteria [60], as well as by Chloroflexi, Fusobacteria, Planctomycetes, and Deltaproteobacteria (Fig 4). Candidate divisions OP8 and JS1 bacteria were absent in the Pink Jello sediment core.

Planctomycetes were present across depth in the Orca Basin slope cores, and Fusobacteria were present in all slope samples except the Sponge Garden core 0–1 cm section. The Pink Jello and Sponge Garden cores shared several major groups, including Nitrospira, Candidate Division SR1, as well as Cyanobacteria, and Alpha, Delta- and Gammaproteobacteria (Fig 4). The Deltaproteobacteria in the Pink Jello core were represented by various uncultured members of the sulfate- and sulfur-reducing families Desulfobacteraceae, Desulfurellaceae and Desulfobulbaceae, as well as unclassified lineages (S1 Fig).

### Archaeal 16S rRNA gene clone libraries

In contrast to highly diverse bacterial lineages, relatively few archaeal lineages were recovered (Fig 4). Most hypersaline brine and sediment clone libraries were dominated by two lineages: the Marine Group-I (MG-I) Thaumarchaeota, a clade at present represented by aerobic, chemolithotrophic ammonia oxidizers that are the predominant archaea in the open-ocean water column [61], and by *Methanohalophilus* spp., a genus of methylotrophic, halotolerant to halophilic methanogens [62]. Within this Orca Basin dataset, MG-I archaea appear to be the only major group that can be potentially linked to nitrification. Other potential nitrifiers were represented only by a single clone from the Sponge garden site; the clone has 95% sequence similarity to *Nitrospina gracilis*, a member of the aerobic, nitrite-oxidizing marine bacterial genus *Nitrospina* within the phylum-level Nitrospinae lineage [63].

The south sub-basin core clone library at 55–60 cm contained one to two sequences each representing sulfate-dependent methane-oxidizing archaea of the ANME-1a, ANME-1b and ANME-2c clusters [64], as well as Marine Group II and Marine Group III archaea within the Thermoplasmatales [65]. In the north sub-basin core 0–5 cm section, a single sequence associated with ANME-2a as well as several sequences associated with Marine Group II were detected. The Pink Jello and Sponge Garden cores yielded mostly MG-I sequences, in addition to a few sequences related to the Deep-Sea Hydrothermal Vent Euryarchaeaotal Group 5 (DHVEG-5), an uncultured lineage containing phylotypes from marine sediments and hypersaline basins [65], and unclassified Thermoplasmatales. Few archaeal clones (N = 5) were recovered from the Red Ridge multicore sediment; these were related to unclassified uncultured Methanomicrobia, Thermoplasmatales and MG-I archaea (Figs 4 and S2).

### Illumina sequencing

**Fig 6:**
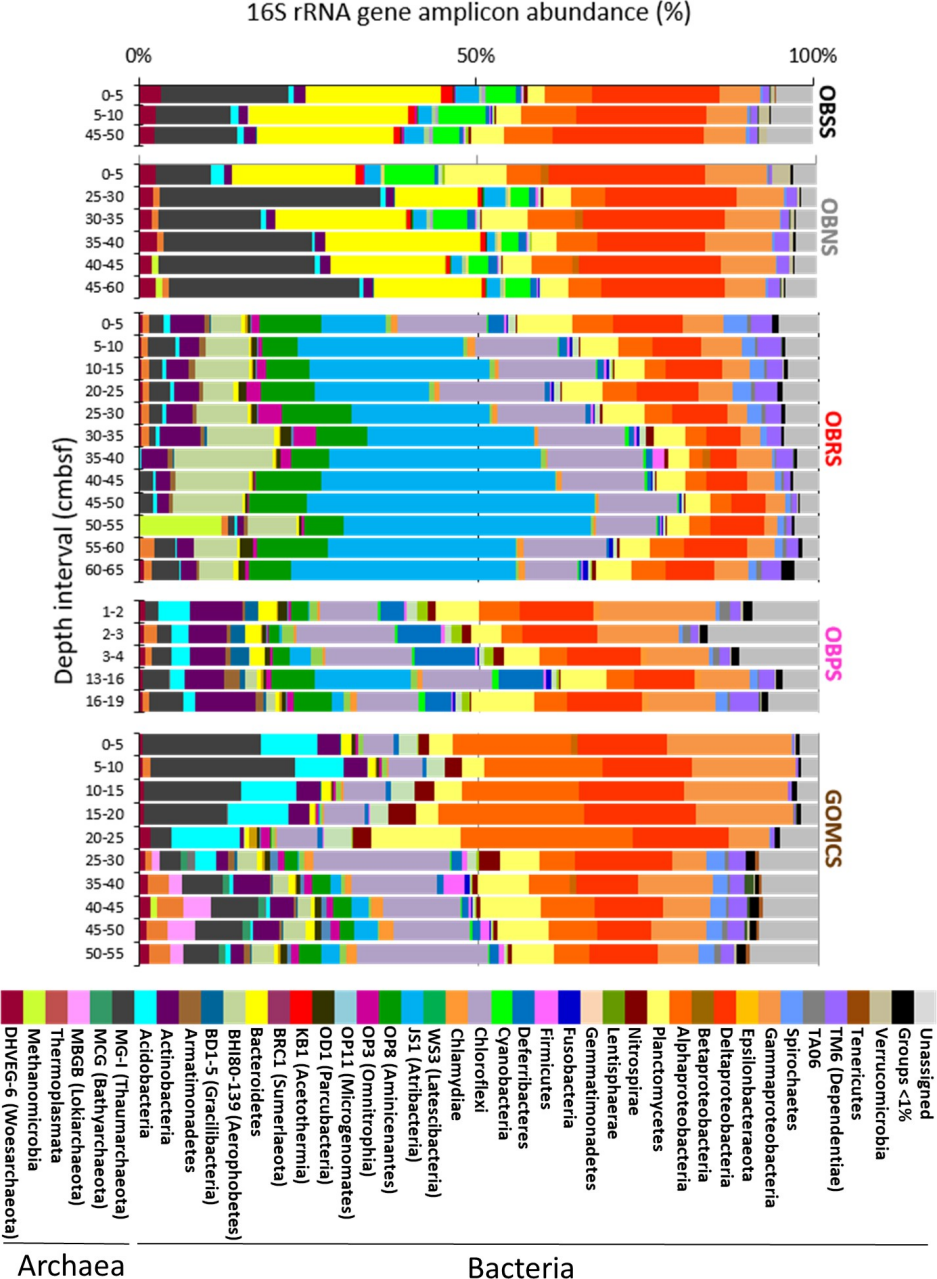
Relative abundance of different taxonomic groups of short (V4) archaeal and bacterial 16S rRNA gene amplicons. Samples include, from top to bottom: OBSS, Orca Basin south sub-basin sediment (MUC-6); OBNS, Orca Basin north sub-basin sediment (MUC-7); OBRS, Orca Basin Red Ridge sediment (MUC-8); OBPS, Orca Basin slope Pink Jello sediment (Alvin core 4560–24); GOMCS, Gulf of Mexico Continental Slope sediment (MUC-13).

A total of 1,338,537 reads were obtained after demultiplexing and quality control filtering (read length 253 bases). After chimera detection, USEARCH OTU clustering, and removal of singletons, 35,488 OTUs were obtained. Rarefaction curves are shown in S3 Fig. The Illumina sequencing survey recovered similar bacterial and archaeal taxa as the clone libraries: 62% (Pink Jello sample, 4650–24 1–2 cm) to 85% (Orca South sediment sample, 0–5 cm) of the Sanger bacterial OTUs were present in the Illumina sequences. For archaeal-related sequences, 70% (Pink Jello sample 4650–24, 16–19 cm) to 100% (MUC 6 Orca south sub-basin sediment sample, 0–5 cm) of the Sanger OTUs were shared with the Illumina sequences amplified from the same sample (S4 Fig). The two amplification and sequencing methods indicated different relative taxon abundances, however, potentially due to known primer biases [66] or sequencing strategy. Differences in relative abundances were especially apparent in the hypersaline samples. Candidate Division KB1 and *Methanohalophilus* appeared as major components of the bacterial and archaeal clone libraries, but accounted only for small fractions (approx. 1% and < 0.5%) among the Illumina sequences of the Orca Basin brine and sediments (Fig 6), except for a localized Methanomicrobia peak in the deepest section of the Red Ridge multicore (S5 Fig). The most frequent OTU (representing between 6–14% of the total sequences) in the Illumina data from the hypersaline sediments was associated with the Bacteroidetes lineage E6aCO2 (S6 Fig), initially detected in a hypersaline endoevaporitic microbial mat [67].

Sequences of the MG-I Thaumarchaeota were found frequently in Orca Basin brine and the sediments collected near the saddle area; they represented the most abundant archaeal group (up to 25% of the total archaeal reads per sample) in almost every Illumina amplicon sample (Fig 6), and also in the archaeal Orca Basin 16S rRNA gene clone libraries, with the sole exception of the ANME-1-rich clone library from the 50–55 cm layer of the north sub-basin (Fig 4). The most frequent Thaumarchaeota-associated OTU was found predominantly in the north sub-basin core, where it accounted for 5–12% of the archaeal reads, while the south sub-basin hypersaline samples contained relative abundances of 2–5% of this OTU. The OTU was highly similar to sequences from hydrothermal fields (e.g. FN553841), and had a BLAST identity of 94% to the 16S rRNA gene from a hypersaline Red Sea Thaumarchaeota genome sequence [68].

Whereas the Illumina sequencing dataset for the hypersaline sediments of Orca Basin were dominated by MG-I archaea, Bacteroidetes, Alpha-, Gamma- and Deltaproteobacteria, the Red Ridge multicore sediment retained these groups in diminished proportions but contained mostly sequences of different phyla (Fig 6), in particular of the candidate phylum Atribacteria [60], the Chloroflexi phylum, the candidate phylum Aminicenantes [59], and the uncultured Aerophobetes (BHI180-139) lineage [69]. These lineages contributed less to the Illumina profiles of the Pink Jello core and the continental slope core, which were instead dominated by Alpha-, Gamma- and Deltaproteobacteria (Fig 6).

Sequences related to sulfate-reducing deltaproteobacteria were detected in both hypersaline cores, and accounted for 3.1 to 6.8 percent of the Illumina dataset. The sequences were phylogenetically similar to cultured halotolerant sulfate-reducing bacteria, including genera and species of the Desulfobulbaceae and Desulfobacteraceae (*Desulfosalsimonas*, *Desulfohalobium utahense*, and *Desulfovermiculus halophilus*) [51–53], or uncultured organisms that are typically found in hypersaline environments [15], for example the deltaproteobacterial MSBL-7 lineage [70] classified by SILVA as a member of the Desulfobacteraceae (S6 and S7 Figs).

Since the Desulfobacteraceae contain the sulfate-reducing syntrophs of sulfate-dependent methane oxidation, the SEEP-SRB1 clade and its subgroups [56), we checked the Illumina datasets for these bacteria. Members of the SEEP-SRB1 bacteria were detected in all cores, although subgroups could not be determined due to insufficient length of the Illumina sequence fragments (S7 Fig). Members of the SEEP-SRB2 clade, an uncultured lineage characteristic of methane seeps and methane–rich sediments [55), were found in low abundance (<1%) in the hypersaline Orca Basin cores. These sequences were present in the Red Ridge core and the slope sediment core (S5 Fig), where they coincided with depth intervals showing higher relative abundances of ANME-1b Methanomicrobia sequences (Figs 6 and S7).

**Fig 7:**
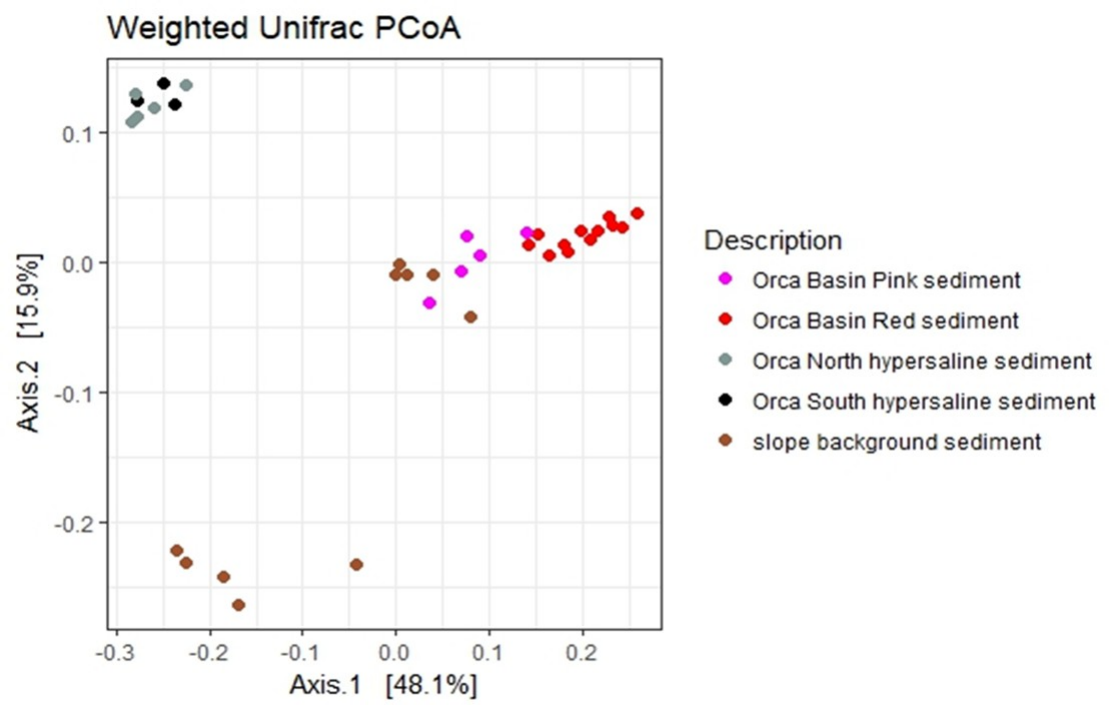
Weighted Unifrac PCoA analyses of Orca Basin Illumina Miseq sequences. The percentage of variation explained by the principle coordinates is plotted on the x (PCo1) and y (PCo2) axis.

Beta diversity patterns for the brine samples indicated potential distinction between some sample types. Weighted UniFrac beta-diversity analysis indicated that north and south sub-basin hypersaline sediment sequences clustered together (Fig 7), while sequences from the Red Ridge core (MUC 8), Pink Jello core, and from the deeper layers (below 25 cmbsf) of the continental slope background core clustered together. The upper sediment layers (above 25 cmbsf) of the background core grouped separately.

## Discussion

The general characteristics of Orca Basin cores (Fig 2) were consistent with previous observations of sediments from the site. The hypersaline sediments at the bottom of Orca Basin show the black color of FeS that is deposited in these Fe-rich sediments under anoxic conditions [14], and the dark-grey bottom layer of the northern basin core were reminiscent of the previously observed interbedded layers of gray open-slope mud that were interpreted as slump deposits from the oxic slope of Orca Basin [3].

The red and pink sediments collected from the saddle area of Orca Basin corresponded to previous descriptions of sediments that are rich in hematite, which is formed under oxic or microaerophilic conditions [13,14]. Porewater salinities of 267 to 261 ppt in the south sub-basin core and 255 to 234 ppt in the north sub-basin core were comparable to previously measured salinities from Deep Sea Drilling Site 618 in the north sub-basin, where the salinity gradually decreased downcore from 237 ppt near the surface towards 48 to 56 ppt at 30 through 90 meters sediment depth [42]. The downcore decline in salinity indicates that the source of the elevated salinity is the overlying brine; in contrast, many shallow brine pools and hypersaline mud volcanoes in the Gulf of Mexico have a subsurface brine source [71]. Na^+^ and Cl^-^ were the major ions responsible for the high salinity in the sediment collected from beneath the brine (Fig 3), consistent with massive halite deposits of the Louann formation dissolving into Orca Basin [2]. The moderate enrichment of K^+^, Ca^2+^ and SO_4_^2-^ relative to seawater was also reported by previous studies of the Orca Basin brine and sediment porewater [1,72]. The elevated sulfate concentrations in the brine were attributed to the dissolution of sulfate-containing salts–possibly anhydrite or gypsum–derived from the Louann halite, whereas the elevated Ca^2+^ and slightly depleted Mg^2+^ concentrations in the southern basin were attributed to dolomitization of calcium carbonate [72].

Brine chemistry shows high variability across different brine basins. While the brines of most DHABs in the Mediterranean Sea and Red Sea as well as Orca Basin, are dominated by NaCl with concentrations ranging from 2.5 to 5.4 mM chloride, the Discovery and Kyros brine basins in the Mediterranean are athalassohaline and contain extremely high (4.4–5.1 mM) concentrations of MgCl_2_. DHABs also differ in sulfide and heavy metal concentrations [15,73]. The brine temperatures of Orca Basin (5°C) [1], Mediterranean brine basins (~14–15°C) [48,74], and most Red Sea brine lakes (22–25°C) are close to the in-situ temperature of overlying seawater, while some Red Sea basins show strong hydrothermal influence, with Discovery Deep and Atlantis II brines reaching 44.8°C and 68°C, respectively [75,76]. Hierarchical clustering of major cation, anion and H_2_S concentrations of brine systems indicated that Orca Basin grouped closest to the Port Sudan brines and several other brine lakes in the Red Sea, and not with Gulf of Mexico brine pool GC233 and mud volcano GB425 [73]. Since distinct physical and chemical characteristics of different brine basins do not match geographical clusters, premature generalizations have to be avoided, and biogeochemistry and microbiology should be considered on a basin-specific basis.

### Comparison of Orca Basin microbial communities to other DHABs

Despite the diversity of environmental conditions, some common brine-related taxa would be expected to be shared between Orca Basin sediments and other hypersaline environments. For example, sequences of KB1 bacteria, a widely distributed and potentially obligately halophilic lineage [44] were found in the hypersaline sediments in this study (Figs 4 and 5), and have been detected consistently in Red Sea DHAB brine and sediments, including Kebrit Deep DHAB sediments [47], and Shaban Deep brine [54], and in Mediterranean Sea brine basins Discovery, L’Atalante, Bannock, Urania [74], Medee [48], Thetis [77],and Kyros [78]. Bacteroidetes were also more abundant in hypersaline Orca Basin sediments, in both Sanger and Illumina sequences (Figs 5 and 6). One of the most abundant OTUs from Orca Basin hypersaline sediments was associated with the uncultured Bacteroidetes E6aCO2 bacteria (Fig 5 NCBI Accession # KR857576; S6 Fig). This uncultured Bacteroidetes group was initially detected in a hypersaline mat [67], and is among the sequences detected in the high MgCl_2_ brine of Kyros Basin [78]. Although Deltaproteobacteria are generally abundant in anoxic sediments, the most abundant deltaproteobacterial sequences in the hypersaline sediments of Orca Basin were specifically associated with sequences detected in other DHABs, including high salinity tolerant members of the Desulfobacteriaceae, Desulfohalobiaceae, and the MBSL group originally detected in Mediterranean DHABs (S1 and S7 Figs) [74], and reported to be in high abundance in other hypersaline basins [78].

However, sequences associated with halophilic bacteria are not always dominant in high salinity deep-sea environments. The hypersaline sediments of Orca Basin contained phylotypes, such as Cyanobacteria, that almost certainly derived from sinking particles that originated in the upper water column and were preserved in the brine as dead cells or extracellular DNA [79]. In the sediments beneath the brine of the Red Sea Atlantis II basin, shallower sediment layers contained taxa typical of the non-brine influenced water column, including SAR11 (pelagic Alphaproteobacteria) and Cyanobacteria; these water column remnants were undetectable only in the deepest sampled layer near 3.5 m sediment depth where identifiable hypersaline taxa were more abundant [80]. In sediments at the edge of Urania, L’Atalante and Discovery basins, sequences related to *Pseudomonas* sp. and other bacteria associated with soil or non-hypersaline sediment dominated over taxa detected in other hypersaline systems, a possible consequence of sediment sampling at the rim of these basins [81,82]. Some sequencing surveys in the Gulf of Mexico have detected non-halophiles more frequently than halophiles. For example, 16S rRNA gene sequences of KB1 sequences were recovered from hypersaline seep sediments at lease block Green Canyon 205, on the Louisiana slope [83], but they were not found in sediments on the edge of a hypersaline shallow brine pool (salinity ~90 ppt) at Alaminos Canyon 601 [84]. Similarly, few sequences related to halophiles were observed from the brine lake at Green Canyon 233 or at 25 cm depth of the hypersaline mud volcano at Garden Banks 425, despite salinities over 125 ppt. Instead, these locations harbored bacterial communities that are shared more broadly by cold seeps and deep-sea sediments, mainly Deltaproteobacteria, Epsilonbacteria, and Gammaproteobacteria, which appeared to reflect proximity to the halocline and to non-halophilic bacterial communities. The low abundance of identifiable halophilic taxa in these environments may also be a consequence of slightly lower salinity, sampling at the periphery of hypersaline habitats, or differences in amplification and/or sequencing strategies.

Among the archaea of Orca Basin, halophiles were represented by halotolerant, methylotrophic methanogens of the genus *Methanohalophilus*, whereas the generally aerobic and heterotrophic Halobacteriales, and the uncultured MSBL1 group (probably sugar-fermenting archaea with additional autotrophic pathways) [85] were not detected. The most dominant archaeal group in both hypersaline and non-hypersaline sediment samples from Orca Basin were the Thaumarchaeota (MG-I archaea) that are common in the marine water column and surficial sediments [61]; these archaea are also found in shallow Gulf of Mexico brine lakes [84]. In the Red Sea DHABs Atlantis II and Discovery Deep, hypersaline sediment cores were dominated by MG-I archaeal pyrotags (73.3 to 96.1%) in almost every sediment section, and their proportional contribution to the total archaea sequence reads decreased only in the deepest and most sulfur-enriched samples in Atlantis II Basin and in Discovery Deep Basin [80]. These data suggest potential accumulation and preservation of MG-I cells and their DNA under hypersaline conditions [79,86]. However, at least some MG-I Thaumarchaeota may have physiological adaptations to hypersaline conditions. A recent study of a *Nitrosopumilus*-related genome analyzed from a Red Sea brine basin (salinity 182 ppt) detected potential osmotolerance genes, specifically ectoine production, glutamate transporters, and a proline-glutamate conversion enzyme [87]. Transcriptional analysis of *Nitrosopumilus maritimus* showed that cells cope with high salinity not only through enhanced synthesis of osmostress-protective ectoines; since the ectoine gene cluster contains a gene for a mechanosensitive channel, they are simultaneously prepared for osmotic down-shock [88]. Transcripts of ammonia monooxygenase have been detected in Mediterranean brine basin sediments, although they have not been linked specifically to MG-I archaea [82]. Organic matter could provide an alternative energy source, as heterotrophic capability was reported for MG-I Thaumarchaeota [89]. As the MG-I archaea demonstrate, the evidence for excluding a microbial group from the halophilic core community is not always straightforward.

### Sulfate, sulfide, and sulfate reduction

While sulfate reduction rates were not measured directly in this study, porewater sulfate profiles and previous measurements indicate potentially low but persistent sulfate-reducing activity in Orca Basin sediments [14,19], which is consistent with our sequencing results detecting members of halophilic sulfate-reducing families and genera (e.g. Desulfohalobiaceae, *Desulfocella*, *Desulfosalsimonas*) (S2 and S8 Figs). Slight downcore decreases of sulfate porewater concentrations, in particular in the north sub-basin core (Fig 3), are consistent with detectable porewater sulfide, and with sulfate reduction rates of 10 to 30 nmol cm^-3^ d^-1^ measured for an Orca Basin north sub-basin core from the same multicorer deployment as this study [19]. Similar potential sulfate reduction rates of 15.97 to 38.43 nmol cm^-3^ d^-1^ were previously measured within the upper 36 cm of the Orca south sub-basin hypersaline sediments, and rates up to 76.59 nmol cm^-3^ d^-1^ were found in the brines [14].

The Orca Basin rates are in the same range as rates from Mediterranean brine lakes (7.80 to 82.15 nmol cm^-3^ d^-1^ [74]; max. 20.4 nmol cm^-3^ d^-1^ in the Urania Basin chemocline [90]). Sulfate reduction extends into deep subsurface sediments of Orca Basin, where it was detected from 4 to 167 m below the sediment surface in Deep Sea Drilling Project Site 619 in the north sub-basin [52,91]. Potential substrates for sulfate-reducing microbial populations–primary alcohols, amino acids and hydrogen—can be inferred based on the substrate spectrum of cultured relatives of deltaproteobacterial phylotypes in Orca Basin, such as the genera *Desulfohalobium* [52] and *Desulfovermiculus* [51]. These Deltaproteobacteria may be more widely distributed and indicate active halophilic populations; for example, In the high MgCl_2_ brine of Kyros basin, sequences related to these deltaproteobacterial groups occurred in high relative abundance where sulfate reduction rates were also highest (603 nmol  cm^-3^ d^−1^) [78]. Although sulfate reduction rates are low in Orca Basin, porewater sulfide would be expected to accumulate over time, as in other geochemically similar brine basins; yet the high concentrations of dissolved ferrous iron that distinguish Orca Basin prevent the accumulation of dissolved sulfides [9,14,72]. The lack of dissolved sulfide, or its presence in very low concentrations, may further attenuate anaerobic microbial processes that depend on sulfide and are commonly observed in sulfidic, fully reduced sediments.

### Dissolved carbon and ammonium

While mud volcanoes and some brine pools have subsurface carbon inputs [71], organic carbon in the Orca Basin brine is likely primarily derived from sinking particles from the water column [7]. The δ^13^C-DIC values of the brine cores cluster near -20‰ throughout the cores (Fig 3) and are consistent with the accumulation of inorganic carbon primarily derived from the remineralization of marine organic matter. The long residence times of particles at the brine-seawater interface may increase the recalcitrance of the organic matter that is ultimately deposited in sediments [11]. Over time, the slow microbial degradation of sedimentary organic matter leads to accumulation of a large pool of sedimentary DOC that exceeds the DOC concentrations in the brine and in non-hypersaline sediments by an order of magnitude (Fig 3). The DOC concentrations measured in the north sub-basin MUC core (5800 μM) were consistent with DOC concentrations at greater depth in Orca Basin subsurface sediments at DSDP Site 618, also located in the north sub-basin [92]. At this DSDP site, the lowest DOC value, 63 μg/ml or 5250 μM, is located at 3 mbsf, followed by the highest DOC peak of 18750 μM at 12 mbsf; the remaining values cluster in the range of 80 to 120 μg/ml or 6600 to 10000 μM and are found in subsurface sediments down to 91 mbsf [92].

Previously measured DOC concentrations of various Gulf of Mexico brine pool and brine flow sediments were observed to be elevated when compared to non-seep or non-hypersaline sediments [71,93]. DOC concentrations in DHAB sediments are reported rarely. In the Atlantis II brine basin, DOC concentrations of 14.9 to 36.5 μg/ml or 1240 to 3042 μM were measured 50 to 250 cmbsf below a barite mound [94]; these concentrations were similar to those (2200 μM) found in the southern basin sediment core of Orca Basin. In general, the high DOC concentrations in hypersaline sediments suggest that DOC accumulates due to slow microbial oxidation degradation and assimilation of DOC. Other degradation products of photosynthetic organic matter, such as ammonium, accumulate as well. High ammonium concentrations in Orca Basin brine and sediments (in the range of 300–500 μM) are congruent with the high ammonium concentrations that are commonly observed in hypersaline brine lakes [84,87,90,95].

### Methane turnover

The final product of anaerobic degradation of organic matter, methane, accumulated in the brine and in sediments in concentrations that suggest some local variability; the previously reported methane concentration of 750 μM in the brine [4] falls between porewater methane concentration of 91 to 156 μM in the south sub-basin and 565 to 1400 μM in the north sub-basin core. The δ^13^C-CH4 values of 75.5 to -76.7‰ in the south sub-basin and -89.6 to -83.1‰ in the north sub-basin are clearly in the biogenic range [41] and are broadly consistent with methylotrophic methanogenesis as the dominant methanogenic pathway [19]. These δ^13^C-CH_4_ values were similar to those (-72.6 to -75.7 ‰) measured previously in deep sediment cores of the north sub-basin (DSDP Site 618), obtained through the Deep-Sea Drilling Project [42]; they are also similar to δ^13^C-CH_4_ values of -73 to 74 ‰ reported for the Orca Basin brine [4]. The slightly more negative δ^13^C-CH_4_ values for the north sub-basin sediments, and the elevated methane concentrations for this basin (Fig 3), indicate greater methanogenic activity in the north sub-basin. To the best of our knowledge, all direct demonstrations of methanogenic activity were so far performed with sediments from the north sub-basin. Methanogenic activity in Orca Basin was shown by incubating subsurface sediments (34 mbsf) from DSDP Site 618 in the north sub-basin with ^14^C-methylamine as methane-producing substrate [91]. Methylotrophic methane production was also found using the substrate ^14^C-methanol in surficial sediments of the north sub-basin; no activity was observed with bicarbonate and acetate [19].

While the available evidence supports methanogenic activity in the Orca Basin brine and sediments, anaerobic methane oxidation appears to be suppressed under hypersaline conditions. Potential AOM rates remained below detection (<1 pmol cm^-3^ d^-1^) in a core from the north sub-basin [19]. A metabolic linkage between sulfate reduction and methane oxidation is not supported; the methane δ^13^C profiles do not indicate the isotopic overprint of methane oxidation, and sequences of ANME archaea and their sulfate-reducing deltaproteobacterial syntrophs [64] appear rarely if at all in clone libraries and Illumina sequencing datasets. Even the most frequent ANME clade, ANME-1, was detected only in very low abundances (<0.01%) in the Illumina sequences of the Orca Basin hypersaline cores (S5 Fig), and sulfate-reducing ANME-associated syntrophs among the Desulfobacteriaceae, the SEEP-SRB1 cluster, were absent (S1 Fig). Few studies have reported on ANME archaea in hypersaline environments. ANME-1 archaea were detected by a 16S rRNA gene survey in a moderately briny, sulfidic methane seep in the Gulf of Mexico [83]; ANME1 and ANME2 archaea were detected in the Atlantis II and Discovery Deep DHABs, predominantly in a sediment sample with high total sulfur content [80]. Overall, Orca Basin represents a seafloor methane trap that accumulates methane without significant diminishment by in-situ anaerobic microbial methane oxidation. A long history of methane accumulation, possibly older than the lifetime of Orca Basin, is consistent with abundant, widely distributed methane hydrates in the sediment column underlying Orca Basin [91].

## Conclusions

The combined geochemical and sequence-based data set indicates that the Orca Basin sediments harbor distinct halophilic bacterial and archaeal communities that participate in several anaerobic biogeochemical processes. Both sequencing and geochemical data indicate sulfate reduction and methanogenesis likely occurs in Orca Basin. In addition to methane, other products of anaerobic microbial decomposition of organic matter accumulate within the Orca Basin sediments and brine, in particular ammonium, DOC, DIC, and reduced metals. As these carbon and energy sources diffuse slowly from the anoxic brine across the halocline and oxycline, they likely sustain a complex microbial ecosystem that thrives on these substrates, and harvests the chemical energy of electron donors that are less energetically advantageous within the anoxic brine and sediment. Future work focusing on a fine-scale survey of the microbial community across the entire chemocline of Orca Basin, from oxic deep-sea water to fully reduced brine, will identify the microbial players and their processes in this complex stratified ecosystem.

## Supporting information

S1 Fig Phylogenetic tree of near-complete Orca Basin deltaproteobacterial 16S rRNA gene clone sequences. (PDF)

S2 Fig Phylogenetic tree of near-complete Orca Basin archaeal 16S rRNA gene clone sequences. (PDF)

S3 Fig Rarefaction analysis of Illumina sequencing observed OTUs. (TIF)

S4 Fig Relative abundance of Sanger bacterial OTUs represented in Illumina sequences. (TIF)

S5 Fig Relative abundance of Methanomicrobia in Orca Basin samples. (TIF)

S6 Fig Phylogenetic tree of most frequent Illumina denovo OTUs from hypersaline sediments. (TIF)

S7 Fig Relative abundance of Deltaproteobacteria in the Illumina dataset. (TIF)

S8 Fig Box plots of alpha diversity estimators. (TIF)

## References

[1] Shokes RF, Trabant PK, Presley BJ, Reid DF. Anoxic, Hypersaline Basin in the Northern Gulf of Mexico. Science. 1977; 196: 1443–6. 10.1126/science.196.4297.1443 17776922

[2] Pilcher RS, Blumstein RD. Brine volume and salt dissolution rates in Orca Basin, northeast Gulf of Mexico. AAPG Bull. 2007; 91: 823–833.

[3] Addy SK, Behrens EW. Time of accumulation of hypersaline anoxic brine in Orca Basin (Gulf of Mexico). Mar Geol. 1980; 37: 241–252.

[4] Wiesenburg DA, Brooks JM, Bernard BB. Biogenic hydrocarbon gases and sulfate reduction in the Orca Basin brine. Geochim Cosmochim Acta. 1985; 49: 2069–80.

[5] Trefry JH, Presley BJ, Keeney-Kennicutt WL, Trocine RP. Distribution and chemistry of manganese, iron, and suspended particulates in Orca Basin. Geo-Mar Lett. 1984; 4: 125–130.

[6] Diercks A, Ziervogel K, Sibert R, Joye SB, Asper V. Vertical Marine Snow Distribution in the Stratified Hypersaline, and Anoxic Orca Basin (Gulf of Mexico). Elem Sci Anthr. 2019; 7:1 10.1525/elementa.348

[7] Shah SR, Joye SB, Brandes JA, McNichol AP. Carbon isotopic evidence for microbial control of carbon supply to Orca Basin at the seawater–brine interface. Biogeosciences. 2013; 10: 3175–83.

[8] Van Cappellen P, Viollier E, Roychoudhury A, Clark L, Ingall E, Lowe K, et al Biogeochemical Cycles of Manganese and Iron at the Oxic−Anoxic Transition of a Stratified Marine Basin (Orca Basin, Gulf of Mexico). Environ Sci Technol. 1998; 32: 2931–9.

[9] Sheu D. D. The Anoxic Orca Basin (Gulf of Mexico)-Geochemistry of Brines and Sediments. Rev Aquat Sci. 1990; 2: 491–507.

[10] Tribovillard N, Bout-Roumazeilles V, Algeo T, Lyons TW, Sionneau T, Montero-Serrano JC, et al Paleodepositional conditions in the Orca Basin as inferred from organic matter and trace metal contents. Mar Geol. 2008; 254: 62–72.

[11] Tribovillard N, Bout-Roumazeilles V, Sionneau T, Serrano JCM, Riboulleau A, Baudin F. Does a strong pycnocline impact organic-matter preservation and accumulation in an anoxic setting? The case of the Orca Basin, Gulf of Mexico. Comptes Rendus Geosci. 2009; 341:1–9.

[12] Harvey HR, Kennicutt MC, others. Selective alteration of Sargassum lipids in anoxic sediments of the Orca Basin. Org Geochem. 1992; 18: 181–187.

[13] Sheu DD, Presley BJ. Formation of hematite in the euxinic Orca Basin, northern Gulf of Mexico. Mar Geol. 1986; 69: 309–321.

[14] Hurtgen MT, Lyons TW, Ingall ED, Cruse AM. Anomalous enrichments of iron monosulfide in euxinic marine sediments and the role of H 2 S in iron sulfide transformations; examples from Effingham Inlet, Orca Basin, and the Black Sea. Am J Sci. 1999; 299:556–88.

[15] Antunes A, Ngugi DK, Stingl U. Microbiology of the Red Sea (and other) deep-sea anoxic brine lakes. Environ Microbiol Rep. 2011; 3:416–33. 10.1111/j.1758-2229.2011.00264.x 23761304

[16] LaRock PA, Lauer RD, Schwarz JR, Watanabe KK, Wiesenburg DA. Microbial biomass and activity distribution in an anoxic, hypersaline basin. Appl Environ Microbiol. 1979; 37: 466–70. 16345355 10.1128/aem.37.3.466-470.1979 PMC243240

[17] Dickins HD, Van Vleet ES. Archaebacterial activity in the Orca Basin determined by the isolation of characteristic isopranyl ether-linked lipids. Deep Sea Res Part II 1992; 39: 521–36.

[18] Van Cappellen P, Viollier E, Roychoudhury A, Clark L, Ingall E, Lowe K, et al Biogeochemical cycles of manganese and iron at the oxic-anoxic transition of a stratified marine basin (Orca Basin, Gulf of Mexico). Environ Sci Technol. 1998; 32:2931–2939.

[19] Zhuang G-C, Elling FJ, Nigro LM, Samarkin V, Joye SB, Teske A, et al Multiple evidence for methylotrophic methanogenesis as the dominant methanogenic pathway in hypersaline sediments from the Orca Basin, Gulf of Mexico. Geochim Cosmochim Acta. 2016; 187: 1–20.

[20] Hoehler TM, Alperin MJ, Albert DB, Martens CS. Field and laboratory studies of methane oxidation in an anoxic marine sediment: Evidence for a methanogen-sulfate reducer consortium. Glob Biogeochem Cycles. 1994; 8: 451–463.

[21] Joye SB, Boetius A, Orcutt BN, Montoya JP, Schulz HN, Erickson MJ, et al The anaerobic oxidation of methane and sulfate reduction in sediments from Gulf of Mexico cold seeps. Chem Geol. 2004; 205: 219–38.

[22] Ertefai TF, Heuer VB, Prieto-Mollar X, Vogt C, Sylva SP, Seewald J, et al The biogeochemistry of sorbed methane in marine sediments. Geochim Cosmochim Acta. 2010; 74: 6033–48.

[23] Crozier TE, Yamamoto S. Solubility of hydrogen in water, sea water, and sodium chloride solutions. J Chem Eng Data. 1974; 19:242–4.

[24] Lin Y-S, Heuer VB, Goldhammer T, Kellermann MY, Zabel M, Hinrichs K-U. Towards constraining H2 concentration in subseafloor sediment: a proposal for combined analysis by two distinct approaches. Geochim Cosmochim Acta. 2012; 77: 186–201.

[25] Cline JD. Spectrophotometric Determination of Hydrogen Sulfide in Natural Waters. Limnol Oceanogr. 1969; 14: 454–8.

[26] Solórzano L. Determination of Ammonia in Natural Waters by the Phenolhypochlorite Method. Limnol Oceanogr. 1969; 14: 799–801.

[27] Teske A, Hinrichs K-U, Edgcomb V, Gomez A de V, Kysela D, Sylva SP, et al Microbial Diversity of Hydrothermal Sediments in the Guaymas Basin: Evidence for Anaerobic Methanotrophic Communities. Appl Environ Microbiol. 2002; 68: 1994–2007. 10.1128/AEM.68.4.1994-2007.2002 11916723 PMC123873

[28] Caporaso JG, Lauber CL, Walters WA, Berg-Lyons D, Huntley J, Fierer N, et al Ultra-high-throughput microbial community analysis on the Illumina HiSeq and MiSeq platforms. ISME J. 2012 6: 1621–4. 10.1038/ismej.2012.8 22402401 PMC3400413

[29] Pruesse E, Peplies J, Glöckner FO. SINA: Accurate high-throughput multiple sequence alignment of ribosomal RNA genes. Bioinformatics. 2012; 28: 1823–9. 10.1093/bioinformatics/bts252 22556368 PMC3389763

[30] Ludwig W, Strunk O, Westram R, Richter L, Meier H, Buchner A, et al ARB: a software environment for sequence data. Nucleic Acids Res. 2004; 32: 1363–71. 10.1093/nar/gkh293 14985472 PMC390282

[31] Caporaso JG, Kuczynski J, Stombaugh J, Bittinger K, Bushman FD, Costello EK, et al QIIME allows analysis of high-throughput community sequencing data. Nat Methods. 2010; 7: 335–6. 10.1038/nmeth.f.303 20383131 PMC3156573

[32] Aronesty E. ea-utils: Command-line tools for processing biological sequencing data. 2011. ea-utils http://code.google.com/p/ea-utils: Expression Analysis, Durham, NC

[33] Edgar RC. UPARSE: highly accurate OTU sequences from microbial amplicon reads. Nat Methods. 2013; 10: 996–8. 10.1038/nmeth.2604 23955772

[34] Pruesse E, Quast C, Knittel K, Fuchs BM, Ludwig W, Peplies J, et al SILVA: a comprehensive online resource for quality checked and aligned ribosomal RNA sequence data compatible with ARB. Nucleic Acids Res. 2007; 35: 7188–96. 10.1093/nar/gkm864 17947321 PMC2175337

[35] Caporaso JG, Bittinger K, Bushman FD, DeSantis TZ, Andersen GL, Knight R. PyNAST: a flexible tool for aligning sequences to a template alignment. Bioinformatics. 2010; 26: 266–7. 10.1093/bioinformatics/btp636 19914921 PMC2804299

[36] Price MN, Dehal PS, Arkin AP. FastTree: Computing Large Minimum Evolution Trees with Profiles instead of a Distance Matrix. Mol Biol Evol. 2009; 26: 1641–50. 10.1093/molbev/msp077 19377059 PMC2693737

[37] Shannon CE. A mathematical theory of communication. Bell Syst Tech J. 1948; 27:379–423, 623–656.

[38] Faith DP. The role of the phylogenetic diversity measure, PD, in bio-informatics: getting the definition right. Evol Bioinformatics Online. 2006; 2: 277–283. PMC2674672 19455221

[39] Hamady M, Lozupone C, Knight R. Fast UniFrac: facilitating high-throughput phylogenetic analyses of microbial communities including analysis of pyrosequencing and PhyloChip data. ISME J. 2009; 4: 17–27. 10.1038/ismej.2009.97 19710709 PMC2797552

[40] McMurdie PJ, Holmes S. phyloseq: an R package for reproducible interactive analysis and graphics of microbiome census data. PloS One. 2013; 8:e61217 10.1371/journal.pone.0061217 23630581 PMC3632530

[41] Whiticar MJ. Carbon and hydrogen isotope systematics of bacterial formation and oxidation of methane. Chem Geol. 1999; 161: 291–314.

[42] Burke RA, Sackett WM, Brooks JM. Hydrogen- and Carbon-Isotope Compositions of Methane from Deep Sea Drilling Project Site 618, Orca Basin. Init Repts DSDP. 1986; 96: 777–780. 10.2973/dsdp.proc.96.148.1986

[43] Sackett WM, Brooks JM, Bernard BB, Schwab CR, Chung H, Parker RA. A carbon inventory for Orca Basin brines and sediments. Earth Planet Sci Lett. 1979; 44: 73–81.

[44] Nigro LM, Hyde AS, MacGregor BJ, Teske A. Phylogeography, Salinity Adaptations and Metabolic Potential of the Candidate Division KB1 Bacteria Based on a Partial Single Cell Genome. Front Microbiol. 2016; 7: 1266; 10.3389/fmicb.2016.01266 27597842 PMC4993014

[45] Isenbarger TA, Finney M, Rios-Velazquez C, Handelsman J, Ruvkun G. Miniprimer PCR, a New Lens for Viewing the Microbial World. Appl Environ Microbiol. 2007; 74: 840–9. 10.1128/AEM.01933-07 18083877 PMC2227730

[46] Kirk Harris J, Gregory Caporaso J, Walker JJ, Spear JR, Gold NJ, Robertson CE, et al Phylogenetic stratigraphy in the Guerrero Negro hypersaline microbial mat. ISME J 2013; 7:50–60 10.1038/ismej.2012.79 22832344 PMC3526174

[47] Eder W, Ludwig W, Huber R. Novel 16S rRNA gene sequences retrieved from highly saline brine sediments of Kebrit Deep, Red Sea. Arch Microbiol. 1999; 172: 213–218. 10.1007/s002030050762 10525737

[48] Yakimov MM, La Cono V, Slepak VZ, La Spada G, Arcadi E, Messina E, et al Microbial life in the Lake Medee, the largest deep-sea salt-saturated formation. Sci Rep. 2013; 3:3554 10.1038/srep03554 24352146 PMC3867751

[49] Brandt KK, Patel BKC, Ingvorsen K. *Desulfocella halophila* gen. nov., sp. nov., a halophilic, fatty-acid-oxidizing, sulfate-reducing bacterium isolated from sediments of the Great Salt Lake. Int J Syst Bacteriol. 1999; 49: 193–200. 10.1099/00207713-49-1-193 10028263

[50] Ollivier B, Hatchikian CE, Prensier G, Guezennec J, Garcia J-L. *Desulfohalobium retbaense* gen. nov., sp. nov., a Halophilic Sulfate-Reducing Bacterium from Sediments of a Hypersaline Lake in Senegal. Int J Syst Bacteriol. 1991; 41: 74–81.

[51] Belyakova EV, Rozanova EP, Borzenkov IA, Tourova TP, Pusheva MA, Lysenko AM, et al The new facultatively chemolithoautotrophic, moderately halophilic, sulfate-reducing bacterium *Desulfovermiculus halophilus* gen. nov., sp. nov., isolated from an oil field. Microbiology. 2006; 75:161–171. 16758868

[52] Jakobsen TF, Kjeldsen KU, Ingvorsen K. *Desulfohalobium utahense* sp. nov., a moderately halophilic, sulfate-reducing bacterium isolated from Great Salt Lake. Int J Syst Evol Microbiol. 2006; 56: 2063–2069. 10.1099/ijs.0.64323-0 16957100

[53] Kjeldsen KU, Jakobsen TF, Glastrup J, Ingvorsen K. Desulfosalsimonas propionicica gen. nov., sp. nov., a halophilic, sulfate-reducing member of the family Desulfobacteraceae isolated from a salt-lake sediment. Int J Syst Evol Microbiol. 2010; 60: 1060–1065. 10.1099/ijs.0.014746-0 19666789

[54] Eder W, Schmidt M, Koch M, Garbe-Schönberg D, Huber R. Prokaryotic phylogenetic diversity and corresponding geochemical data of the brine–seawater interface of the Shaban Deep, Red Sea. Environ Microbiol. 2002; 4: 758–763. 10.1046/j.1462-2920.2002.00351.x 12460284

[55] Knittel K, Boetius A, Lemke A, Eilers H, Lochte K, Pfannkuche O, et al Activity, Distribution, and Diversity of Sulfate Reducers and Other Bacteria in Sediments above Gas Hydrate (Cascadia Margin, Oregon). Geomicrobiol J. 2003; 20: 269–94.

[56] Schreiber L, Holler T, Knittel K, Meyerdierks A, Amann R. Identification of the dominant sulfate-reducing bacterial partner of anaerobic methanotrophs of the ANME-2 clade. Environ Microbiol. 2010; 12: 2327–40. 10.1111/j.1462-2920.2010.02275.x 21966923

[57] Kleindienst S, Ramette A, Amann R, Knittel K. Distribution and in situ abundance of sulfate-reducing bacteria in diverse marine hydrocarbon seep sediments. Environ Microbiol. 2012; 14: 2689–2710. 10.1111/j.1462-2920.2012.02832.x 22882476

[58] Lösekann T, Knittel K, Nadalig T, Fuchs B, Niemann H, Boetius A, et al Diversity and abundance of aerobic and anaerobic methane oxidizers at the Haakon Mosby Mud Volcano, Barents Sea. Appl Env Microbiol. 2007; 73: 3348–62. 17369343 10.1128/AEM.00016-07 PMC1907091

[59] Farag IF, Davis JP, Youssef NH, Elshahed MS. Global Patterns of Abundance, Diversity and Community Structure of the Aminicenantes (Candidate Phylum OP8). PLoS ONE. 2014; 9:e92139 10.1371/journal.pone.0092139 24637619 PMC3956909

[60] Nobu MK, Dodsworth JA, Murugapiran SK, Rinke C, Gies EA, Webster G, et al Phylogeny and physiology of candidate phylum “Atribacteria” (OP9/JS1) inferred from cultivation-independent genomics. ISME J. 2016 10:273–86. 10.1038/ismej.2015.97 26090992 PMC4737943

[61] Pester M, Schleper C, Wagner M. The Thaumarchaeota: an emerging view of their phylogeny and ecophysiology. Curr Opin Microbiol. 2011; 14:300–6. 10.1016/j.mib.2011.04.007 21546306 PMC3126993

[62] Guan Y, Ngugi DK, Vinu M, Blom J, Alam I, Guillot S, et al Comparative genomics of the genus Methanohalophilus, including a newly isolated strain from Kebrit deep in the Red Sea. Front Microbiol. 2019; 10.839; 10.3389/fmicb.2019.00839 PMC6491703 31068917

[63] Lücker S, Nowka B, Rattei T, Spieck E, Daims H. The genome of *Nitrospina gracilis* illuminates the metabolism and evolution of the major marine nitrite oxidizer. Front Microbiol. 2013; 4:27 10.3389/fmicb.2013.00027 23439773 PMC3578206

[64] Knittel K, Boetius A. Anaerobic Oxidation of Methane: Progress with an Unknown Process. Annu Rev Microbiol. 2009; 63: 311–34. 10.1146/annurev.micro.61.080706.093130 19575572

[65] Durbin AM, Teske A. Sediment-associated microdiversity within the Marine Group I Crenarchaeota: Environ Microbiol Rep. 2010; 2: 693–703. 10.1111/j.1758-2229.2010.00163.x 23766257

[66] Parada AE, Needham DM, Fuhrman JA. Every base matters: assessing small subunit rRNA primers for marine microbiomes with mock communities, time series and global field samples. Environ Microbiol. 2016; 18: 1403–14. 10.1111/1462-2920.13023 26271760

[67] Sørensen KB, Canfield DE, Teske AP, Oren A. Community Composition of a Hypersaline Endoevaporitic Microbial Mat. Appl Environ Microbiol. 2005; 71: 7352–65. 10.1128/AEM.71.11.7352-7365.2005 16269778 PMC1287706

[68] Ngugi DK, Blom J, Alam I, Rashid M, Ba-Alawi W, Zhang G, et al Comparative genomics reveals adaptations of a halotolerant thaumarchaeon in the interfaces of brine pools in the Red Sea. ISME J. 2015; 9: 396–411. 10.1038/ismej.2014.137 25105904 PMC4303633

[69] Ben Hania W, Ghodbane R, Postec A, Brochier-Armanet C, Hamdi M, Fardeau M-L, et al Cultivation of the first mesophilic representative (“mesotoga”) within the order Thermotogales. Syst Appl Microbiol. 2011; 34:581–5. 10.1016/j.syapm.2011.04.001 21596510

[70] Pachiadaki MG, Yakimov MM, LaCono V, Leadbetter E, Edgcomb V. Unveiling microbial activities along the halocline of Thetis, a deep-sea hypersaline anoxic basin. ISME J. 2014; 8:2478–89. 10.1038/ismej.2014.100 24950109 PMC4260694

[71] Joye SB, Bowles MW, Samarkin VA, Hunter KS, Niemann H. Biogeochemical signatures and microbial activity of different cold-seep habitats along the Gulf of Mexico deep slope. Deep Sea Res Part II 2010; 57: 1990–2001.

[72] Schijf J. Alkali elements (Na, K, Rb) and alkaline earth elements (Mg, Ca, Sr, Ba) in the anoxic brine of Orca Basin, northern Gulf of Mexico. Chem Geol. 2007; 243: 255–74.

[73] Merlino G, Barozzi A, Michoud G, Ngugi DK, Daffonchio D. Microbial ecology of deep-sea hypersaline anoxic basins. FEMS Microbiol Ecol. 2018; 94:fiy085. 10.1093/femsec/fiy085 29905791

[74] van der Wielen PWJJ, Bolhuis H, Borin S, Daffonchio D, Corselli C, Giuliano L, et al The enigma of prokaryotic life in deep hypersaline anoxic basins. Science. 2005; 307: 121–3. 10.1126/science.1103569 15637281

[75] Hartmann M. Atlantis-II Deep geothermal brine system. Chemical processes between hydrothermal brines and Red Sea deep water. Mar Geol. 1985; 64: 157–177.

[76] Swift SA, Bower AS, Schmitt RW. Vertical, horizontal, and temporal changes in temperature in the Atlantis II and Discovery hot brine pools, Red Sea. Deep Sea Res Part I 2012; 64: 118–28.

[77] La Cono V, Smedile F, Bortoluzzi G, Arcadi E, Maimone G, Messina E, et al Unveiling microbial life in new deep-sea hypersaline Lake Thetis. Part I: Prokaryotes and environmental settings. Environ Microbiol. 2011; 13: 2250–68. 10.1111/j.1462-2920.2011.02478.x 21518212

[78] Steinle L, Knittel K, Felber N, Casalino C, de Lange G, Tessarolo C, et al Life on the edge: active microbial communities in the Kryos MgCl2 -brine basin at very low water activity. ISME J. 2018; 12:1414–26. 10.1038/s41396-018-0107-z 29666446 PMC5956074

[79] Borin S, Crotti E, Mapelli F, Tamagnini I, Corselli C, Daffonchio D. DNA is preserved and maintains transforming potential after contact with brines of the deep anoxic hypersaline lakes of the Eastern Mediterranean Sea. Saline Syst. 2008; 4:10 10.1186/1746-1448-4-10 18681968 PMC2531117

[80] Siam R, Mustafa GA, Sharaf H, Moustafa A, Ramadan AR, Antunes A, et al Unique Prokaryotic Consortia in Geochemically Distinct Sediments from Red Sea Atlantis II and Discovery Deep Brine Pools. PLoS ONE. 2012; 7:e42872 10.1371/journal.pone.0042872 22916172 PMC3423430

[81] Kormas KA, Pachiadaki MG, Karayanni H, Leadbetter ER, Bernhard JM, Edgcomb VP. Inter-comparison of the potentially active prokaryotic communities in the halocline sediments of Mediterranean deep-sea hypersaline basins. Extremophiles. 2015; 19: 949–60. 10.1007/s00792-015-0770-1 26174531

[82] Edgcomb VP, Pachiadaki MG, Mara P, Kormas KA, Leadbetter ER, Bernhard JM. Gene expression profiling of microbial activities and interactions in sediments under haloclines of E. Mediterranean deep hypersaline anoxic basins. ISME J. 2016; 10: 2643–57. 10.1038/ismej.2016.58 27093045 PMC5113848

[83] Lloyd KG, Lapham L, Teske A. An anaerobic methane-oxidizing community of ANME-1b archaea in hypersaline Gulf of Mexico sediments. Appl Environ Microbiol. 2006; 72:7218–7230. 10.1128/AEM.00886-06 16980428 PMC1636178

[84] Crespo-Medina M, Bowles MW, Samarkin VA, Hunter KS, Joye SB. Microbial diversity and activity in seafloor brine lake sediments (Alaminos Canyon block 601, Gulf of Mexico). Geobiology. 2016; 14: 483–498. 10.1111/gbi.12185 27444236

[85] Mwirichia R, Alam I, Rashid M, Vinu M, Ba-Alawi W, Anthony Kamau A, et al Metabolic traits of an uncultured archaeal lineage -MSBL1- from brine pools of the Red Sea. Sci Rep. 2016; 13;6: 19181 10.1038/srep19181 26758088 PMC4725937

[86] Danovaro R, Corinaldesi C, Dell’Anno A, Fabiano M, Corselli C. Viruses, prokaryotes and DNA in the sediments of a deep-hypersaline anoxic basin (DHAB) of the Mediterranean Sea. Environ Microbiol. 2005; 7: 586–92. 10.1111/j.1462-2920.2005.00727.x 15816935

[87] Kamanda Ngugi D, Blom J, Alam I, Rashid M, Ba-Alawi W, Zhang G, et al Comparative genomics reveals adaptations of a halotolerant thaumarchaeon in the interfaces of brine pools in the Red Sea. ISME J. 2015; 9: 396–411. 10.1038/ismej.2014.137 25105904 PMC4303633

[88] Widderich N, Czech L, Elling FJ, Könneke M, Stöveken N, Pittelkow M, et al Strangers in the archaeal world: osmostress-responsive biosynthesis of ectoine and hydroxyectoine by the marine thaumarchaeon Nitrosopumilus maritimus. Environ Microbiol. 2016; 18:1227–48. 10.1111/1462-2920.13156 26636559

[89] Li M, Baker BJ, Anantharaman K, Jain S, Breier JA, Dick GJ. Genomic and transcriptomic evidence for scavenging of diverse organic compounds by widespread deep-sea archaea. Nat Commun. 2015; 6:8933 10.1038/ncomms9933 26573375 PMC4660358

[90] Borin S, Brusetti L, Mapelli F, D’Auria G, Brusa T, Marzorati M, et al Sulfur cycling and methanogenesis primarily drive microbial colonization of the highly sulfidic Urania deep hypersaline basin. Proc Natl Acad Sci. 2009; 106: 9151–9156. 10.1073/pnas.0811984106 19470485 PMC2685740

[91] Whelan JK, Oremland R, Tarafa M, Smith R, Howarth R, Lee C. Evidence for sulfate-reducing and methane-producing microorganisms in sediments from sites 618, 619 and 622. Init Repts DSDP 1986; 96: 768–75.

[92] Ishizuka T, Kawahata H, Aoki S. Interstitial water geochemistry and clay mineralogy of the Mississippi fan and Orca and Pigmy basins, Deep Sea Drilling Project Leg 96. Init Repts DSDP. 1986; 96: 711–728.

[93] Bowles M, Hunter KS, Samarkin V, Joye S. Patterns and variability in geochemical signatures and microbial activity within and between diverse cold seep habitats along the lower continental slope, Northern Gulf of Mexico. Deep Sea Res Part II Top Stud Oceanogr. 2016; 129:31–40.

[94] Wang Y, Li JT, He LS, Yang B, Gao ZM, Cao HL, et al Zonation of Microbial Communities by a Hydrothermal Mound in the Atlantis II Deep (the Red Sea). PLoS ONE. 2015; 10:e0140766 10.1371/journal.pone.0140766 26485717 PMC4613831

[95] Joye SB, Samarkin VA, MacDonald IR, Hinrichs K-U, Elvert M, Teske AP, et al Metabolic variability in seafloor brines revealed by carbon and sulphur dynamics. Nat Geosci. 2009; 2:349–354.

