# Supplementary material for: Microbial ecology and biogeochemistry of hypersaline sediments in Orca Basin
Source: PLoS One. 2020 Apr 21;15(4):e0231676. doi: 10.1371/journal.pone.0231676 (PMC7173876; doi:10.1371/journal.pone.0231676)
Supplement: S1 Fig — (PDF) [file pone.0231676.s001.pdf]

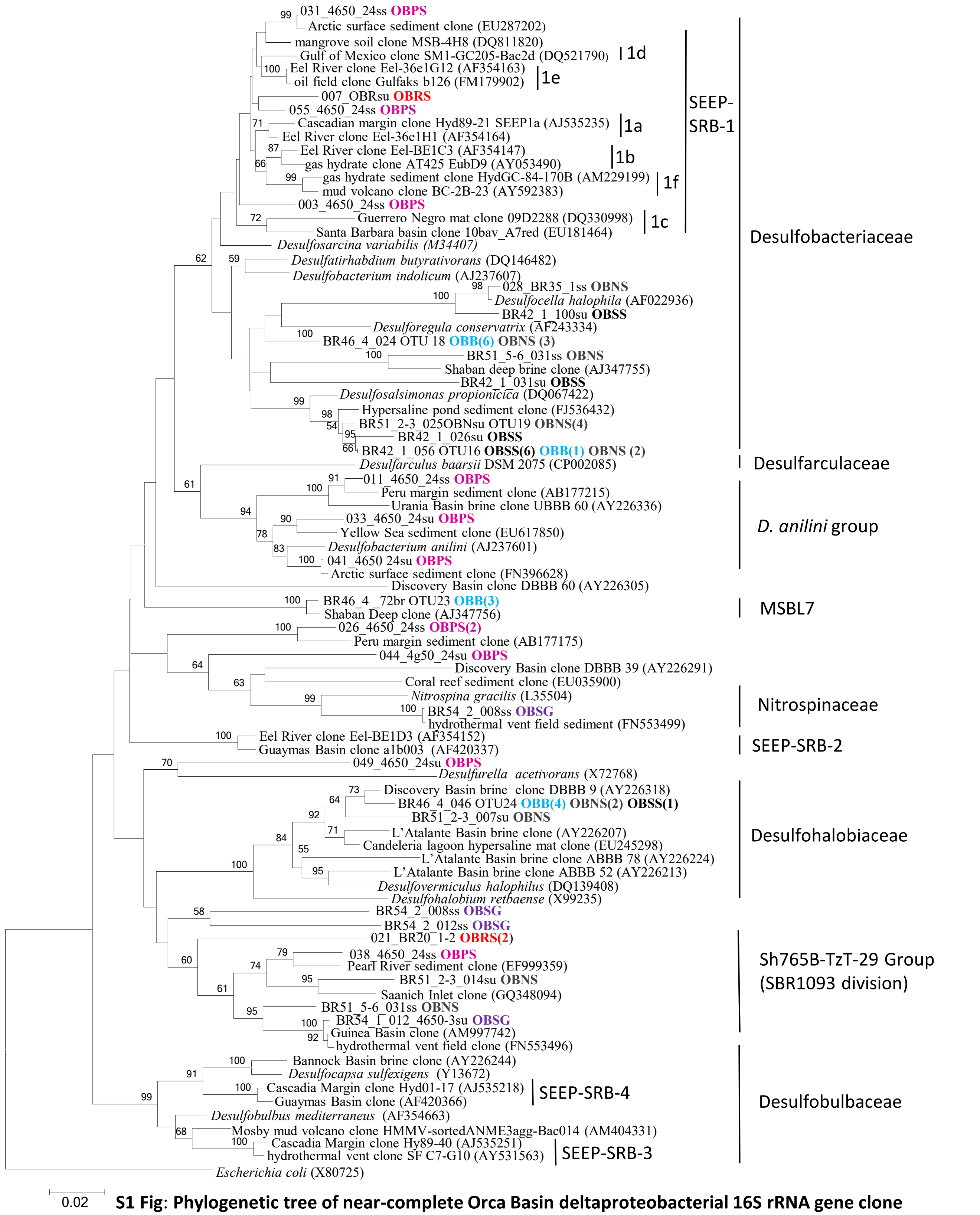

S1 Fig: **Phylogenetic tree of near-complete Orca Basin deltaproteobacterial 16S rRNA gene clone sequences.** Tree was constructed with the NJ method. Bootstrap values are displayed next to nodes. Only bootstrap values of >50% shown. OBB: Orca basin south sub-basin brine. **OBSS**: Orca Basin south sub-basin hypersaline sediment (MUC 6), **OBNS**: Orca Basin north sub-basin hypersaline sediment (MUC 7), **OBRS**: Orca Basin saddle area red sediment (MUC 8), **OBPS**: Orca Basin saddle area pink sediment (core 4650-24). **OBSG**: Orca Basin saddle area sponge garden (core 4650-3) For sites with multiple sequences > 97% , number of clone sequences per OTU are indicated in parentheses.
