# Supplementary figures and images for: Microbial ecology and biogeochemistry of hypersaline sediments in Orca Basin
Source: PLoS One. 2020 Apr 21;15(4):e0231676. doi: 10.1371/journal.pone.0231676 (PMC7173876; doi:10.1371/journal.pone.0231676)

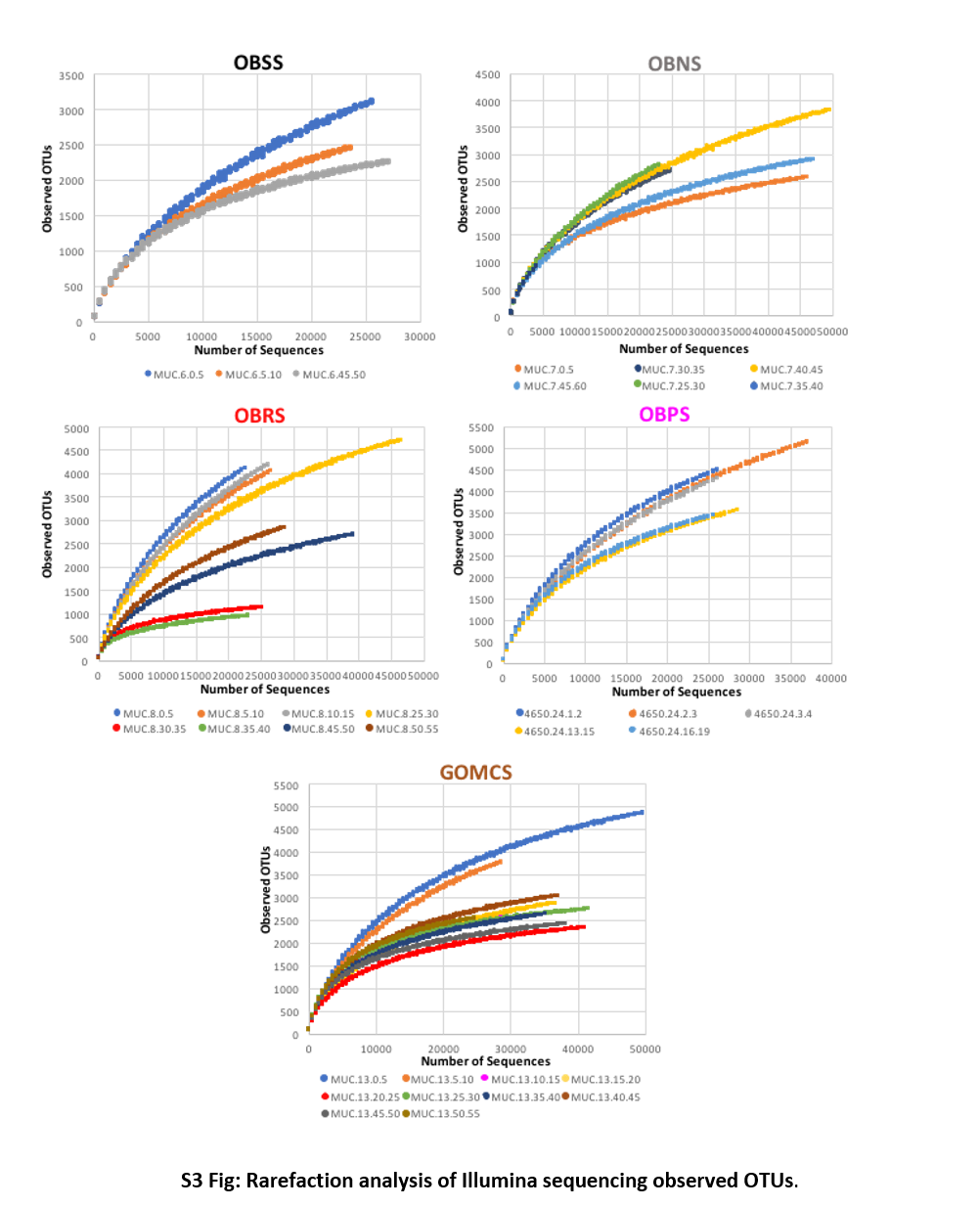

Supplement: S3 Fig — (TIF) [file pone.0231676.s003.tif]

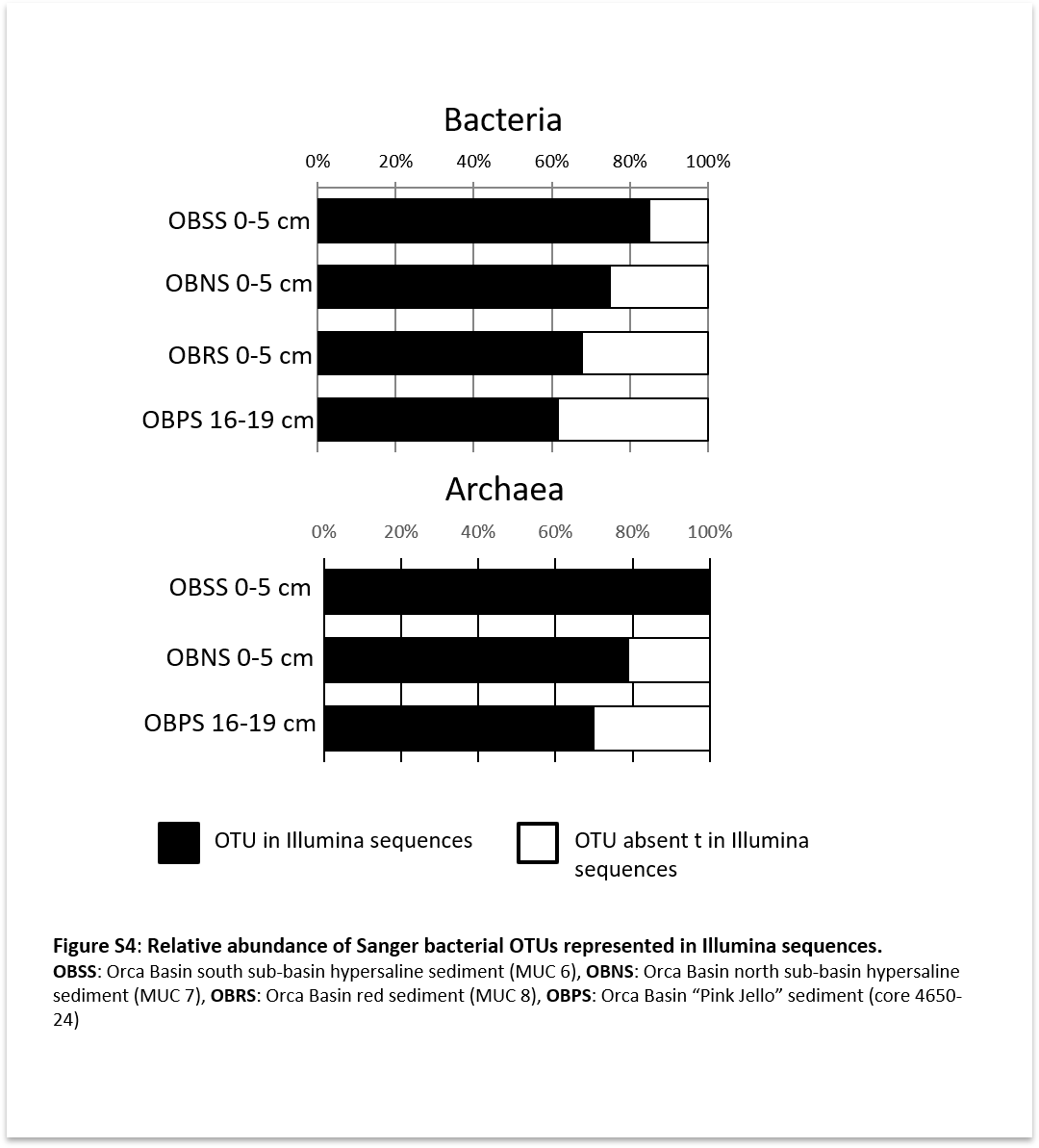

Supplement: S4 Fig — (TIF) [file pone.0231676.s004.tif]

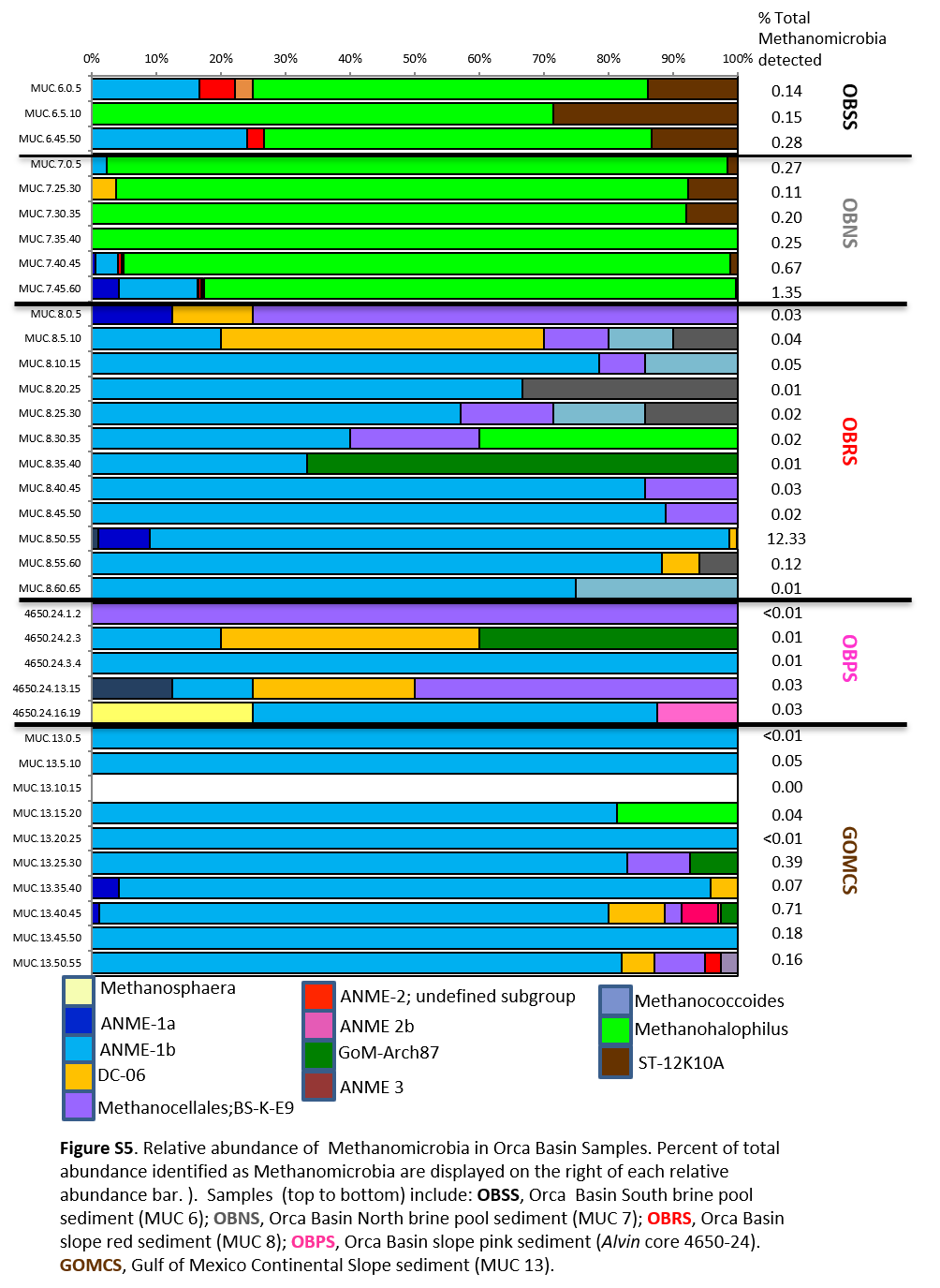

Supplement: S5 Fig — (TIF) [file pone.0231676.s005.tif]

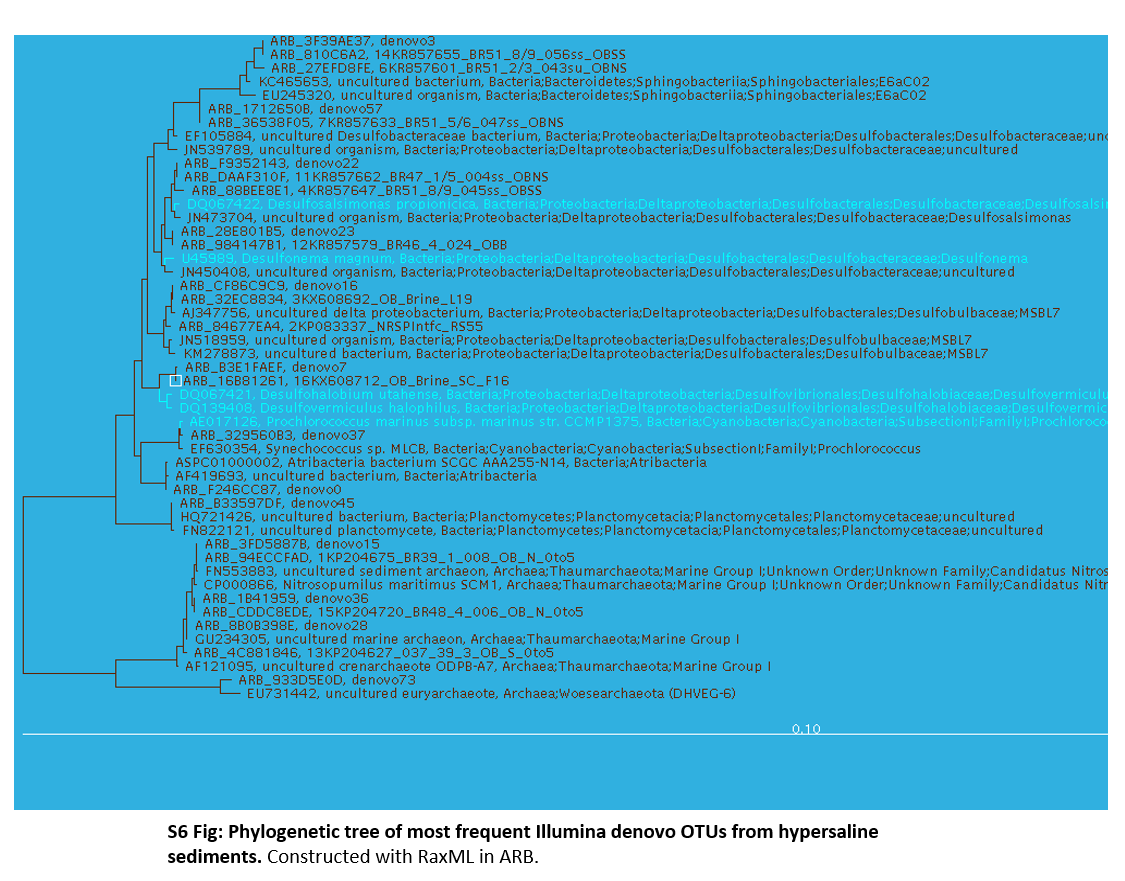

Supplement: S6 Fig — (TIF) [file pone.0231676.s006.tif]

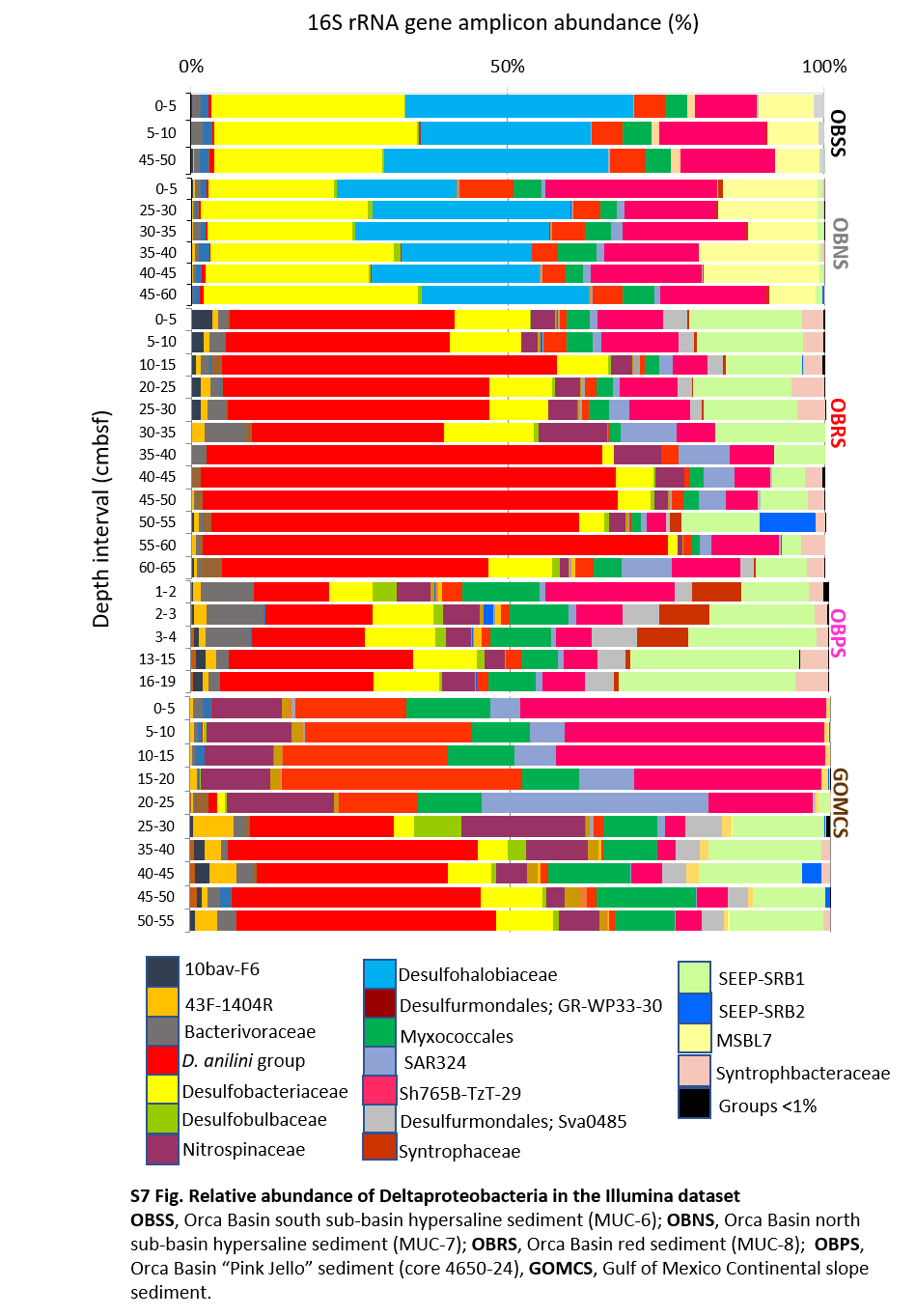

Supplement: S7 Fig — (TIF) [file pone.0231676.s007.tif]

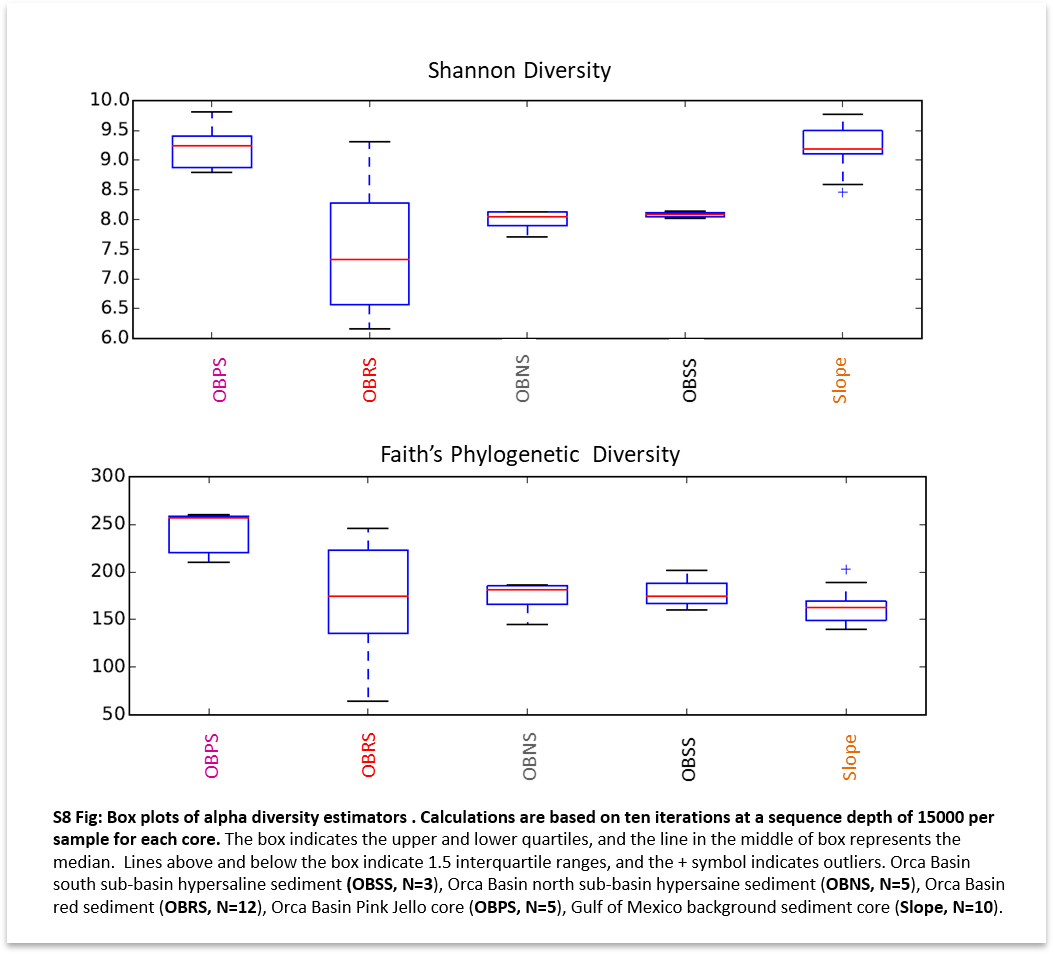

Supplement: S8 Fig — (TIF) [file pone.0231676.s008.tif]
